# Cell type-specific expression profiling unravels the development and evolution of stinging cells in sea anemone

**DOI:** 10.1186/s12915-018-0578-4

**Published:** 2018-09-27

**Authors:** Kartik Sunagar, Yaara Y Columbus-Shenkar, Arie Fridrich, Nadya Gutkovich, Reuven Aharoni, Yehu Moran

**Affiliations:** 1 Department of Ecology, Evolution and Behavior, Alexander Silberman Institute of Life Sciences, The Hebrew University of Jerusalem, 9190401 Jerusalem, Israel; 2 Evolutionary Venomics Lab, Centre for Ecological Sciences, Indian Institute of Science, Bangalore, 560012 India

**Keywords:** Cnidocyte transcriptome, Cnidocyst, Cnidaria, *Nematostella vectensis*, Cell type-specific transcriptome, Cnido-Jun

## Abstract

**Background:**

Cnidocytes are specialized cells that define the phylum Cnidaria. They possess an “explosive” organelle called cnidocyst that is important for prey capture and anti-predator defense. An extraordinary morphological and functional complexity of the cnidocysts has inspired numerous studies to investigate their structure and development. However, the transcriptomes of the cells bearing these unique organelles are yet to be characterized, impeding our understanding of the genetic basis of their biogenesis.

**Results:**

In this study, we generated a nematocyte reporter transgenic line of the sea anemone *Nematostella vectensis* using the CRISPR/Cas9 system. By using a fluorescence-activated cell sorter (FACS), we have characterized cell type-specific transcriptomic profiles of various stages of cnidocyte maturation and showed that nematogenesis (the formation of functional cnidocysts) is underpinned by dramatic shifts in the spatiotemporal gene expression. Among the genes identified as upregulated in cnidocytes were Cnido-Jun and Cnido-Fos1—cnidarian-specific paralogs of the highly conserved c-Jun and c-Fos proteins of the stress-induced AP-1 transcriptional complex. The knockdown of the cnidocyte-specific c-Jun homolog by microinjection of morpholino antisense oligomer results in disruption of normal nematogenesis.

**Conclusions:**

Here, we show that the majority of upregulated genes and enriched biochemical pathways specific to cnidocytes are uncharacterized, emphasizing the need for further functional research on nematogenesis. The recruitment of the metazoan stress-related transcription factor c-Fos/c-Jun complex into nematogenesis highlights the evolutionary ingenuity and novelty associated with the formation of these highly complex, enigmatic, and phyletically unique organelles. Thus, we provide novel insights into the biology, development, and evolution of cnidocytes.

**Electronic supplementary material:**

The online version of this article (10.1186/s12915-018-0578-4) contains supplementary material, which is available to authorized users.

## Background

Cnidocytes, also known as stinging cells, are specialized neural cells that typify the phylum Cnidaria (sea anemones, corals, hydroids, and jellyfish) [1–3]. These cells contain an organelle called cnida or cnidocyst, which is the product of extensive Golgi secretions. Cnidae are composed of a complex capsule polymer characterized by cysteine-rich peptides, such as minicollagens and nematocyst outer wall antigen (NOWA) [1, 4]. Further, the capsule elongates at its end into a tubule that is made up of a polymer of peptides, including minicollagens, nematogalectins, and other structural proteins [5, 6]. This tubule invaginates into the capsule during maturation and remains tightly coiled until activated during prey capture or defense, which results in the discharge of the cnidocyte capsule and uncoiling of the tubule. Cnidae are arguably the morphologically most complex organelles found in nature, and their explosive discharge is one of the fastest biomechanical processes recorded in the animal kingdom [1, 7].

Cnidocysts can be divided into three main categories: (i) nematocysts, the dart-shaped cnidae with spines on hollow tubules that are used for prey piercing and venom injection; (ii) spirocysts, the elastic cnidae used for prey entanglement in Anthozoa; and (iii) ptychocysts which are found exclusively in the cerianthid sea anemones. Nematocysts can be further divided into 25 subcategories based on their morphology [2]. Such an extraordinary morphological and functional complexity of cnidocysts has driven several genetic and biochemical studies striving to identify the molecular mechanisms underlying the formation of this enigmatic organelle [8–10]. However, no study to date has uncovered the complete transcriptomic landscape of cnidocytes, impeding our understanding of the evolutionary origin and the molecular mechanisms underpinning the maturation of these complex organelles.

Here, we characterize the cnidocyte transcriptome of the sea anemone *Nematostella vectensis*, a rising model organism for the study of cnidarian biology [3, 11]. Using the Clustered Regularly Interspaced Short Palindromic Repeats (CRISPR)/Cas9 system [12, 13], we genetically engineered transgenic lines that express a fluorescent protein in their cnidocytes. We then proteolytically dissociated tissues and separated the resulting fluorescent cnidocytes from the other types of cells using a fluorescence-activated cell sorter (FACS). This led to the characterization and identification of thousands of genes that are differentially expressed at various stages of cnidocyte maturation. Our findings shed light on the remarkable complexity and the dynamics of nematogenesis (cnidocyst biogenesis). This experimental design further enabled the identification of cnidocyte-specific transcription factors that have originated in Cnidaria from a metazoan stress-related transcription factor complex around 500 million years ago. By genetically manipulating *Nematostella*, we demonstrate the role of these unique transcription factors in nematogenesis, particularly towards the formation of an integral capsule protein. Thus, our study provides novel insights into the fascinating biology and the development of cnidocytes—the first venom delivery apparatus to appear in animals.

## Results

### Generating a cnidocyte reporter line

**Fig. 1:**
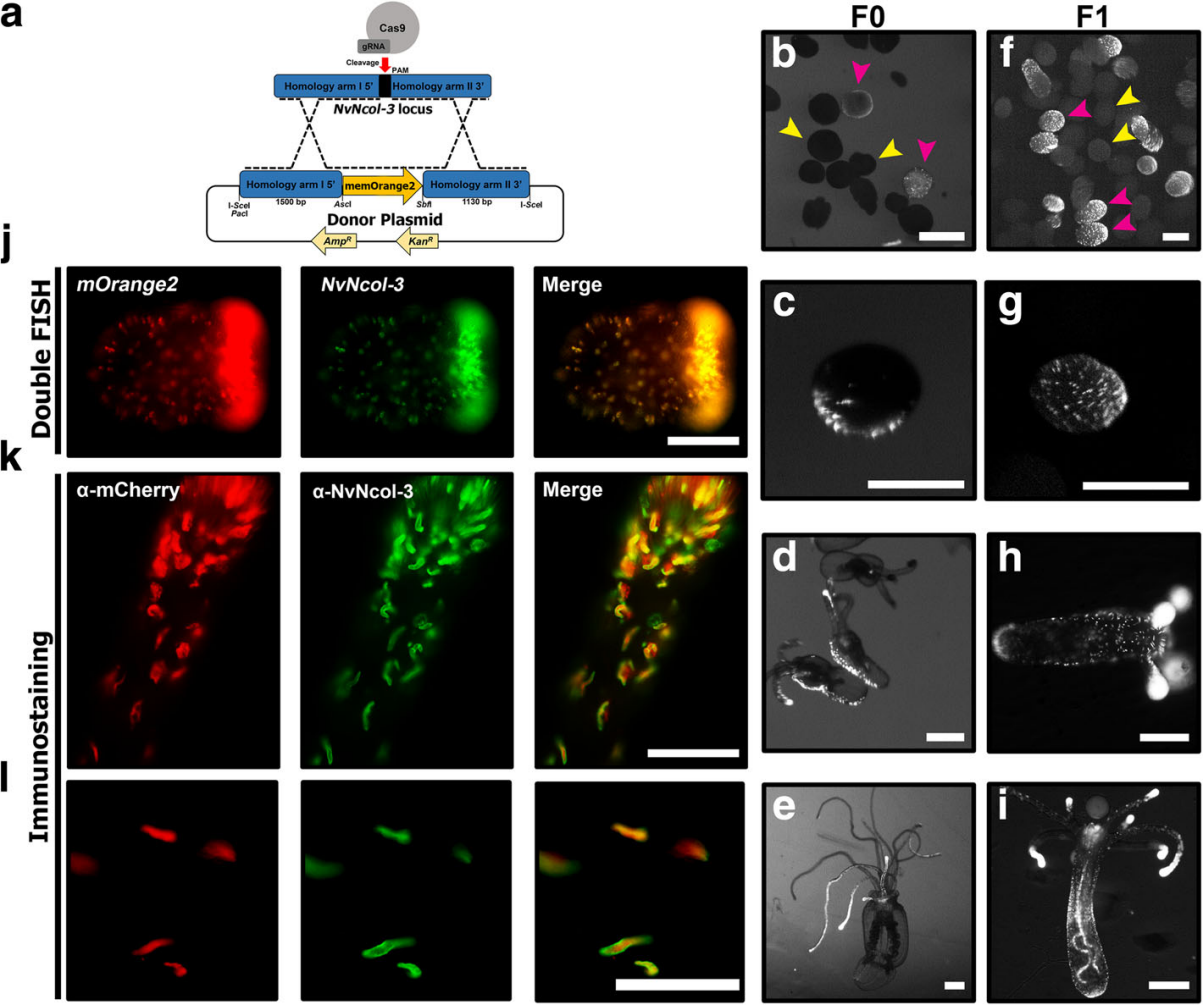
Cnidocyte reporter line. **a** The depiction of the reporter construct used for the generation of the transgenic reporter line. **b**, **c** Expression of memOrange2 in planulae of F0 (injected) and F1 (first filial generation) (**f**–**g**). Magenta arrowheads point towards examples for positive animals, whereas yellow arrowheads point towards examples for negative animals. Scale bar is 250 μm. **d**, **h** Expression of memOrange2 in F0 and F1 primary polyps. Scale bar is 250 μm. **e**, **i** Expression of memOrange2 in F0 and F1 juvenile polyps (2.5 months old). Scale bar is 1000 μm. **j**, **k** RNA expression and protein localization of memOrange2 and NvNcol-3, revealed by dFISH and immunostaining, in early planulae and tentacles. **l** A higher magnification capture of immunostaining in a tentacle performed as in **k**. Scale bar in (**j**) is 100 μm, 50 μm in **k**, and 25 μm in **l**

To generate a transgenic line that expresses a fluorescent reporter protein in its cnidocytes, we used the CRISPR/Cas9 system to knock the reporter gene into the genomic locus of the minicollagen *NvNcol-3*, which is a major structural protein of the cnidocyst capsule wall and, hence, a well-characterized cnidocyte marker in *Nematostella* [14]. The reporter gene encodes mOrange2 [15] with a C-terminal RAS-derived membrane tag (hereinafter referred to as memOrange2). We followed a similar approach that was described before in *Nematostella* [16], with the only exception being the absence of promoter and regulatory sequences in the donor construct (Fig. 1a). The microinjected embryos started expressing the fluorescent protein in their cnidocytes 72 h post fertilization (hpf) (Fig. 1b, c). Of 2633 injected zygotes, 1053 survived the procedure, and of them, 314 exhibited by 72 hpf a signal detectable by a fluorescent stereomicroscope. As expected of F0 *Nematostella* embryos, the transgene expression was restricted to small-to-medium patches [17], a phenomenon that was also documented in primary polyps and juvenile stages (Fig. 1d, e). The second generation (F1) exhibited specific and strong expression of memOrange2 in a very wide population of cnidocytes at the planulae stage (Fig. 1f, g), with extremely strong expression in the tentacles of the primary polyp and juvenile stages (Fig. 1h, i). Our utilization of the CRISPR/Cas9 system exploits the homologous recombination-based DNA repair mechanism of the cell to insert the transgene into the native gene and, thus, places the transgene under the control of its native regulatory elements. Hence, the expression pattern of the reporter gene accurately mirrored the native expression of *NvNcol-3*, both at the mRNA and protein levels, as demonstrated by double fluorescent in situ hybridization (dFISH) and immunostaining experiments (Fig. 1j–l), respectively. In the immunostaining experiment, 85–100% of the cells were positive for both antibodies (a mean overlap of 93%), indicating an overlapping expression. Only a small minority of cells expressed either NvNcol-3 or memOrange2 without an overlap.

### Isolating distinct cnidocyte populations

**Fig. 2:**
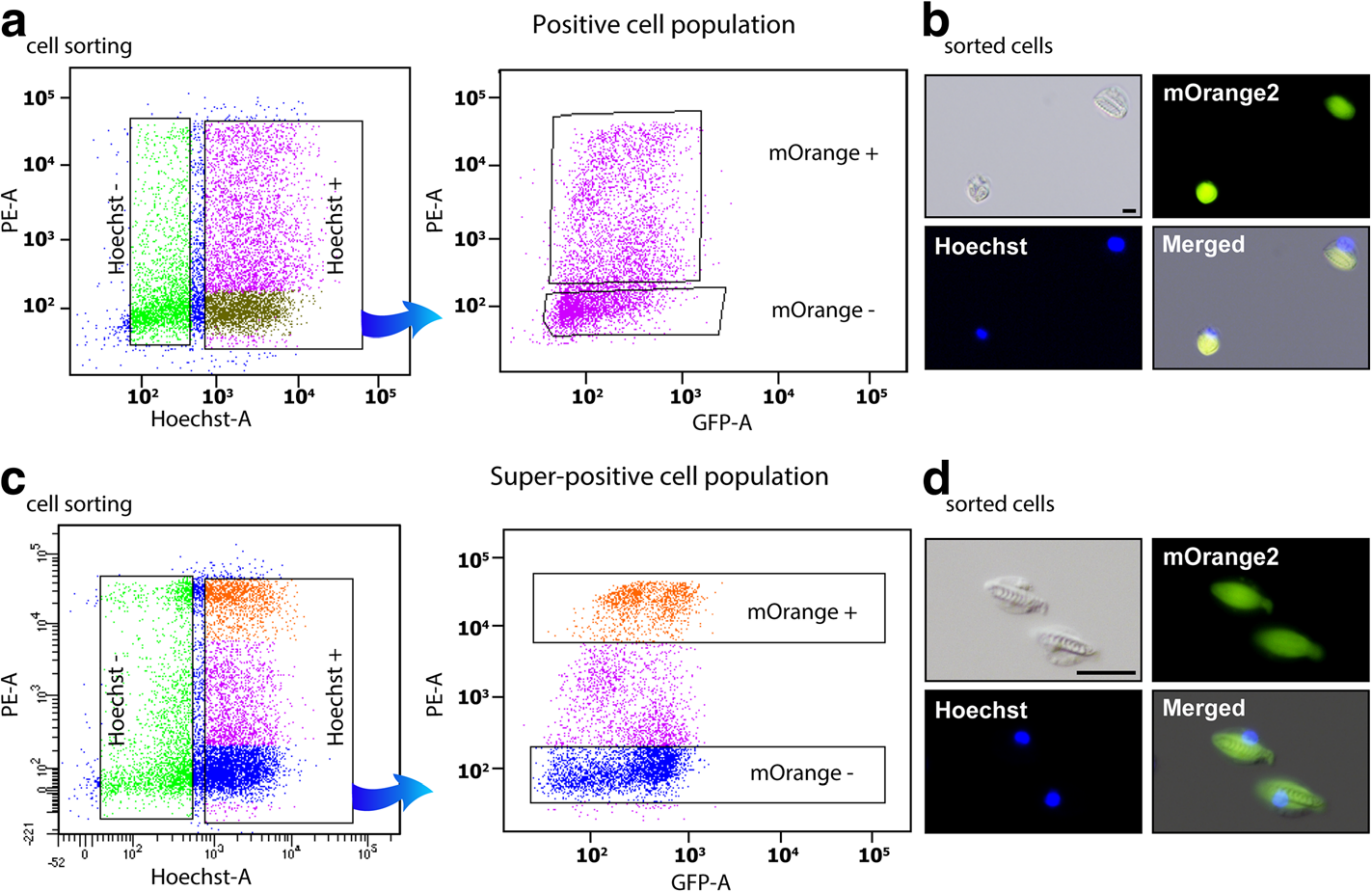
Distinct cnidocyte populations. Panels **a** and **c** highlight the FACS-assisted sorting strategies employed in this study. The cells are first sorted into Hoechst-negative and Hoechst-positive populations, followed by the separation of the latter into memOrange2-positive, memOrange2-super-positive, and memOrange2-negative populations. Panels **b** and **d** depict images of sorted positive and super-positive cells, as observed in differential interference contrast (DIC), mOrange, and DAPI filters, respectively, under a fluorescent microscope. A merger of these three images is also shown. Scale bar is 20 μm in **b** and **d**

We dissociated tentacles of ten juvenile polyps of the *NvNcol-3*::*memOrange2* transgenic line (3–4 months old; ~ 50 tentacles in total) and sorted their cells according to the intensity of fluorescence. We initially collected two different cell populations: one that included all cells that were positive for Hoechst stain (indication of an intact nucleus) and memOrange2 (hereinafter referred to as “positive cells”) and another population that was positive for Hoechst but negative for memOrange2 (“negative cells”) (Fig. 2a). Then, we employed a similar strategy to collect the third population of cells that were not only positive for Hoechst but expressed very high levels of memOrange2 as well (“super-positive cells”) (Fig. 2c). Like before, we also collected negative cells for comparison (Hoechst positive but memOrange2 negative). Microscopic inspection of fluorescent cells revealed that while the positive cells included many young developing cnidocytes, which are characterized by small undeveloped capsules or very few memOrange2-filled vesicles (Fig. 2b; Additional file 1: Figure S1), the super-positive cell population was largely enriched with mature cnidocytes that were easily identifiable due to the presence of elongated capsules (Fig. 2d; Additional file 1: Figure S1). To verify that memOrange2-positive cells are indeed young cnidocytes and that their fluorescence follows the expression pattern of NvNcol-3, we fixed and co-immunostained dissociated cells. Indeed, these cells were found to be positive for both memOrange2 and NvNcol-3 (Additional file 2: Figure S2). We isolated mRNA from the aforementioned cell populations in triplicates and employed high-throughput sequencing to characterize gene expression patterns.

### Characterizing maturation stage-specific cnidocyte transcriptomes

**Fig. 3:**
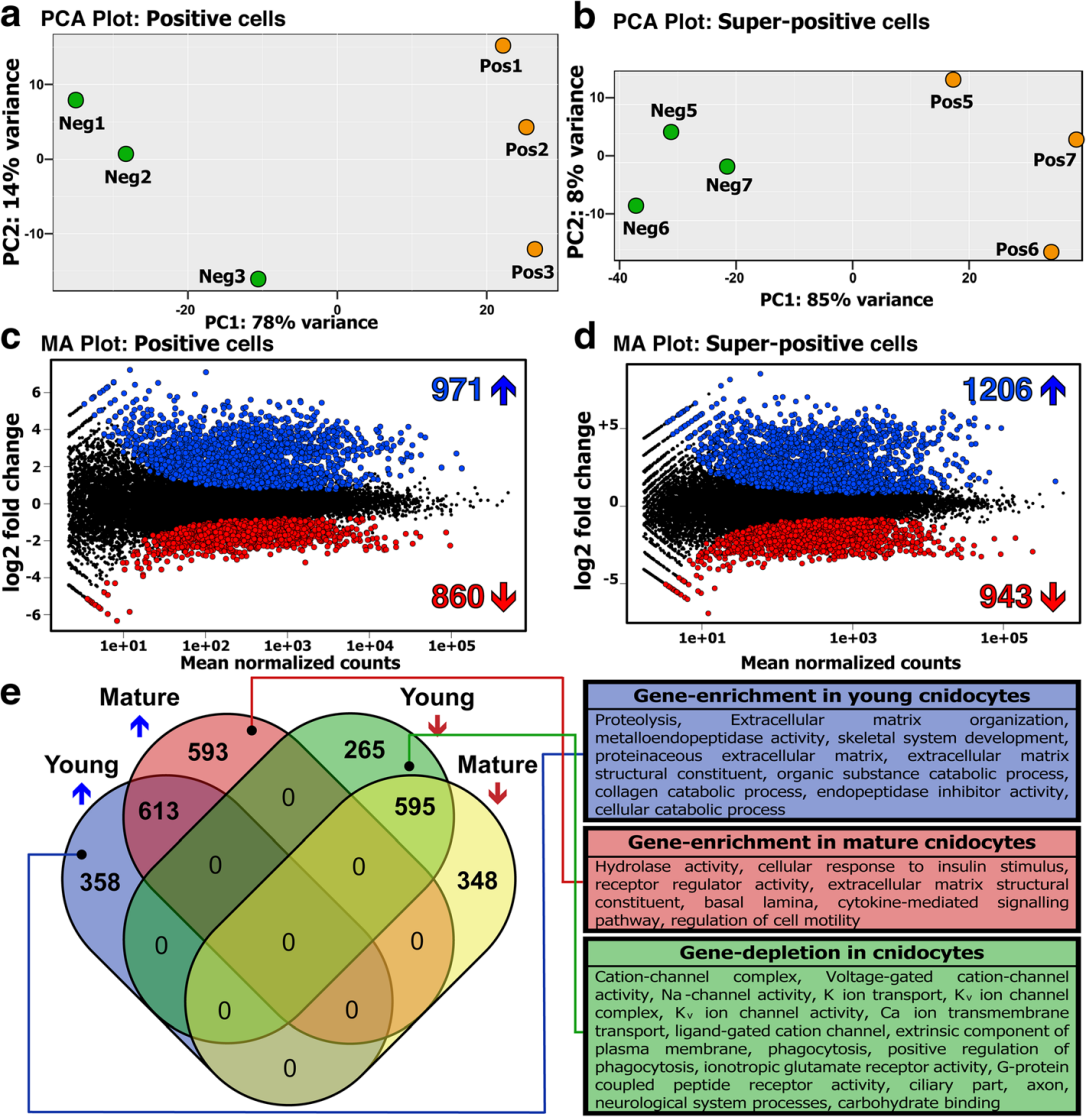
Differentially expressed genes within cnidocytes. Panels (**a**) and (**b**) show the clustering of biological replicates (memOrange2 negative: Neg1–3 and Neg5–7, positive: Pos1–3, and super-positive cell populations: Pos5–8) in a principal component analysis, where the axes represent the first two principal components, labeled with the percentages of variance associated with each axis. Panels (**c**) and (**d**) show MA-plots, which represent the log ratio of differential expression as a function of the mean intensity for each feature. The total number of upregulated and downregulated genes, highlighted in the plot as blue and red circles, respectively, is also indicated. Panel (**e**) depicts a Venn diagram, showing a comparison of differentially expressed genes identified in the positive and super-positive populations. The total number of genes uniquely identified as either upregulated or downregulated in positive and super-positive cells can be attributed to early-stage and mature cnidocytes, respectively. Enriched and depleted annotation features of each of these cell populations are also indicated

To characterize maturation stage-specific expression profiles of cnidocytes, we employed two different comparisons: (i) positives vs negatives and (ii) super-positives vs negatives. The principal component analysis revealed distinct gene expression profiles of the biological conditions (positives or super-positives vs their respective negatives) and uniform expression of biological replicates within the respective condition (Fig. 3a, b). Differential expression analyses by two different methods in concert (DESeq2 and edgeR) enabled the stringent identification of a large number of differentially expressed genes in positive and super-positive cell populations, in comparison to the negative cells (Additional file 3: Figure S3). Our analyses revealed the significant upregulation of genes encoding known cnidocyte markers, such as *NvNcol-1*, *NvNcol-4*, *NEP-3*, *NEP-3-like*, *NEP-4*, and *NEP-5* [14, 18, 19] in positive cells (Additional file 4: Figure S4). Furthermore, certain genes that are known to lack expression in cnidocytes [20–22], such as the neuronal markers *FMRFamide* and *ELAV*—the latter only identified by DESeq2—were found to be downregulated in both the positive and super-positive cells, in comparison to the negative cells (Additional file 4: Figure S4; Additional file 5: Figure S5). These results demonstrate the robustness of our experimental approach in isolating cnidocytes and accurately characterizing their transcriptional profiles. However, failing to identify cnidocyte markers as differentially expressed in the super-positive cells, despite their upregulation in positive cells, was surprising (Additional file 4: Figure S4; Additional file 5: Figure S5). Moreover, the transcripts encoding memOrange2 and NvNcol-3 were not identified as significantly upregulated in both positive and super-positive cnidocytes.

A total of 1831 and 2149 differentially expressed genes were identified in common by DESeq2 and edgeR in positive and super-positive cell populations, respectively (Fig. 3, d; Additional file 6: Table S1). Overall, 613 and 595 genes were commonly identified as upregulated or downregulated, respectively, in the positive and super-positive cell populations, in comparison to the negative cells (Fig. 3e). Interestingly, 358 and 265 genes were identified as significantly upregulated and downregulated, respectively, in the positive cell population alone. These differentially expressed genes, identified only in the positive cells, represent genes that are either upregulated or downregulated during the earlier stages of cnidocyte maturation but not in the mature cnidocyte-rich super-positive population. Similarly, we identified 593 and 348 genes as uniquely upregulated and downregulated, respectively, in the mature cnidocytes of the super-positive population but not in the earlier stages of maturation (Fig. 3e).

### Spatiotemporal dynamics of cnidocyte transcripts and proteins

**Fig. 4:**
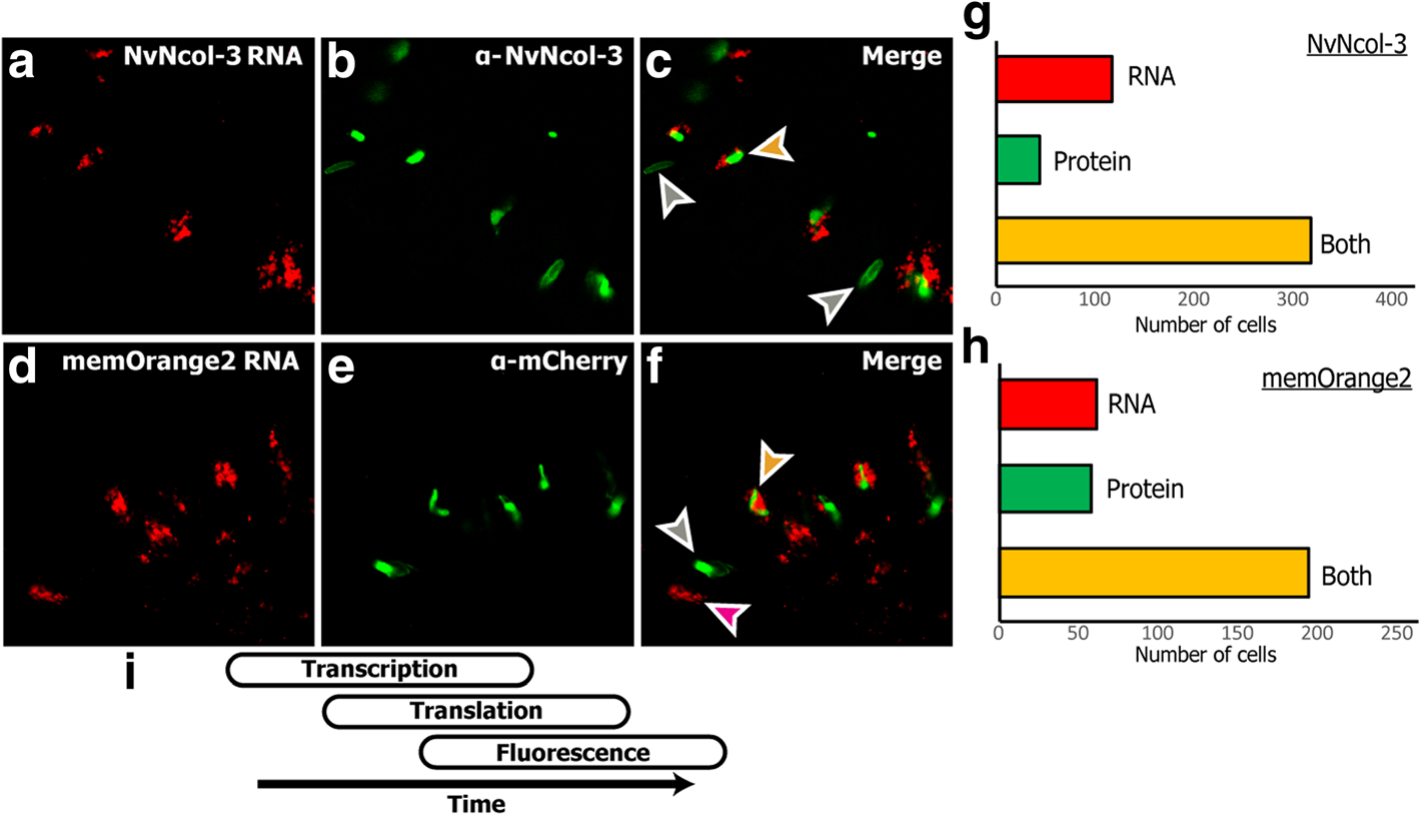
Variability between the transcriptional and proteomic expression levels of cnidocyte-expressed genes. This figure shows that the expression patterns of *NvNcol-3* and *memOrange2* transcripts stained in FastRed (panels **a** and **d**) differ from the expression pattern of their protein products stained with Alexa fluor 488 (panels **b** and **e**). Examples for cells containing only RNA, only protein or both RNA and protein are indicated by magenta, gray and orange arrowheads, respectively (panels **c** and **f**). Quantifications of the cells positive for only RNA, only protein, or both RNA and protein (each from seven animals) are shown as column graphs (panels **g** and **h**). **i** A schematic diagram shows the overlap between the times of gene expression, protein secretion, and protein maturation. Scale bars are 10 μm

The absence of significant enrichment of *NvNcol-3* and *memOrange2* transcripts in both the positive and super-positive cell populations was surprising. We suspected that this might be connected to the maturation stage of the isolated cnidocyte and that, perhaps, the temporal differences in expression levels of *memOrange2* mRNA and protein may result in an inability to capture very early-stage cnidocytes. To test this hypothesis, we performed an ISH experiment, followed by immunostaining. These combined assays enabled us to distinguish between *NvNcol-3* mRNA and protein expression in the 3-day-old wild-type planulae (Fig. 4a–c) and between the mRNA and the protein of the marker gene *memOrange2* in the transgenic planulae (Fig. 4d–f).

This experiment revealed spatiotemporal differences in the mRNA and protein expression patterns of both these genes within cnidocytes. In both the wild-type and *NvNcol-3* transgenic planulae, a significant proportion of cnidocytes exclusively stained either at the mRNA level or at the protein level (Fig. 4a–h). In seven wild-type planulae, we observed 117 cells that were positive only to *NvNcol-3* RNA, 44 cells that were positive only for the antibody, and 319 cells that were positive for both (Fig. 4g). In seven transgenic planulae, we detected 62 cells that were positive only to *memOrange2* RNA, 58 cells that were positive only for the antibody, and 195 cells that were positive for both (Fig. 4h). The cells that only expressed mRNA lacked capsules or contained undeveloped capsules and multiple vesicles and are probably cnidoblasts or early-stage cnidocytes (Fig. 4), whereas those containing only protein housed fully developed or nearly mature capsules (Fig. 4; Additional file 1: Figure S1). Only a subset of the cnidocytes, most of which were characterized by relatively small and immature capsules—typical of developing cnidocytes—exhibited expression at both mRNA and protein levels (Fig. 4). These findings are in agreement with the recently reported co-localization experiments of NvNcol-3 [23].

### Discovery of novel genes with cnidocyte-specific expression

**Fig. 5:**
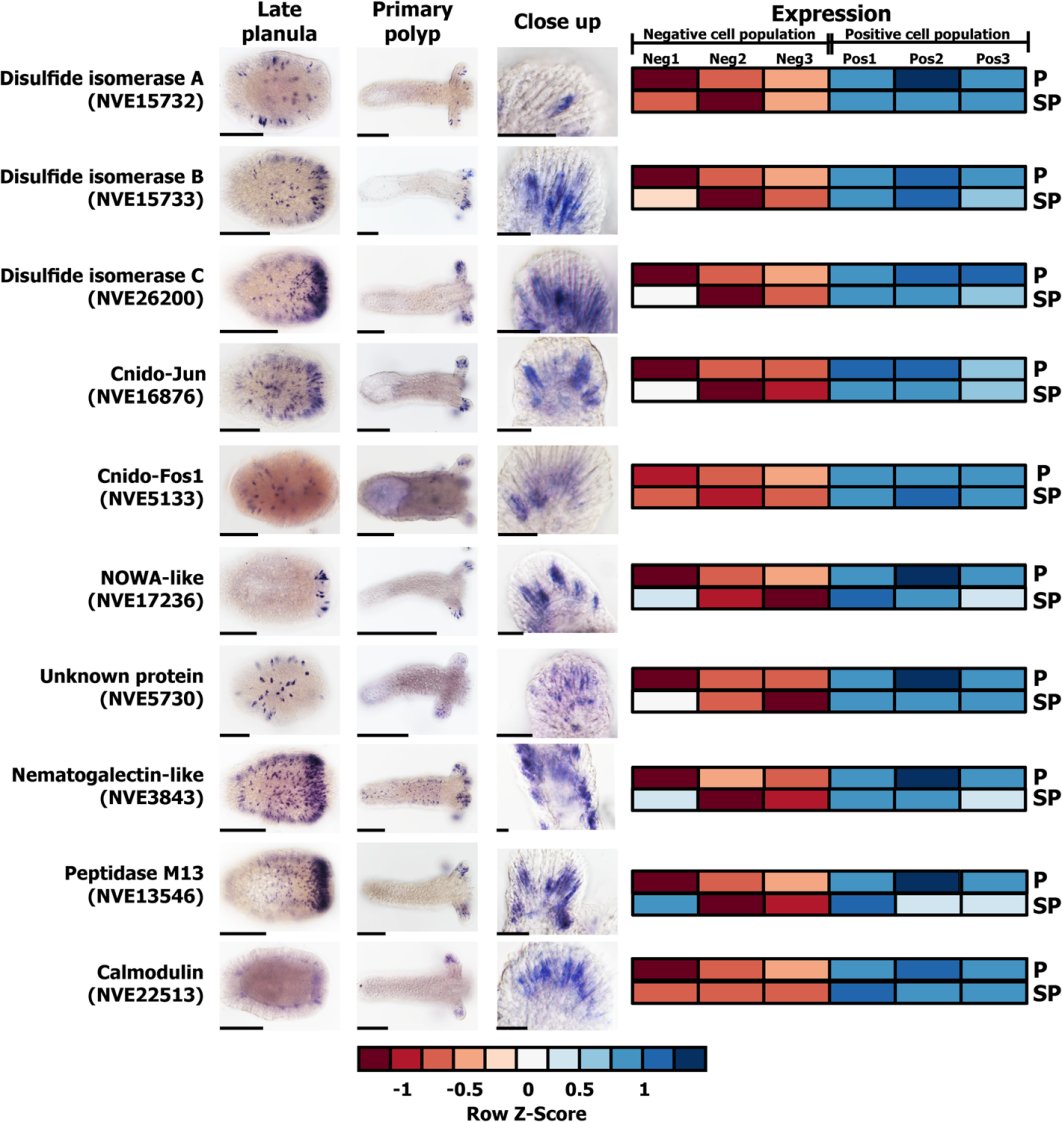
Novel genes with cnidocyte-specific expression. In situ hybridization expression patterns of novel cnidocyte-specific genes identified in this study are shown in late planula and primary polyp. The elongated cells in the ectoderm, seen in the closeup image, can clearly be identified as cnidocytes according to the large unstained space, which is the cnidocyst capsule. Heatmaps of expression levels of each gene in positive (P) and super-positive (SP) cell populations, each across three technical replicates (positives and super-positive indicated as Pos1–3 and negatives as Neg1–3) are also provided. A color code for expression values, ranging from a gradient of red (downregulated) to blue (upregulated), is depicted at the bottom. Scale bars are 100 μm for planulae and primary polyps and 20 μm for closeup panels

One of the major objectives of this study was to identify novel genes with cnidocyte-specific expression, as this would advance our knowledge regarding the cnidocyte biology, their genetic basis of development, and evolutionary origin. First, to test the accuracy of our experimental and bioinformatic approaches for identifying differentially expressed genes in cnidocytes, we chose ten genes that were found in our current analyses to be significantly upregulated in positive cells (log_2_ fold change in the range of 2.8 to 5) (Fig. 5). Interestingly, nine of these ten genes were not previously described in the cnidocytes of *Nematostella*. They include genes that encode protein disulfide isomerases (*NVE15732*, *NVE15733*, and *NVE26200*), a homolog of the c-Jun transcription factor (*NVE16876*) that we named “Cnido-Jun,” a homolog of the c-Fos transcription factor (*NVE5133*) that we named “Cnido-Fos1,” a highly derived homolog of NOWA (*NVE17236*), an M13 peptidase (*NVE13546*), a calmodulin (*NVE22513*), an unknown protein containing thrombospondin and F5/8 type C domains (*NVE5730*), and a protein containing a galactose-binding lectin domain with an apparent homology to nematogalectins (*NVE3843*), a family of structural proteins found in cnidocytes [6, 23]. The localization of expression using ISH highlighted the cnidocyte-specific expression of these ten genes (Fig. 5). Unexpectedly, the expression pattern of individual genes exhibited a distinct spatiotemporal variability (Fig. 5). For example, the expression of the NOWA-like gene was limited to very few cnidocytes in the oral region of the planula larva and the tentacles of the primary polyp, while the expression of Cnido-Jun was localized to both the oral and the central part of the planula, followed by an increased expression in the tentacles of the primary polyp (Fig. 5). The three protein disulfide isomerases also showed noticeably distinct expression patterns, strongly indicating functional specialization of these enzymes (Fig. 5). To validate the cnidocyte-specific expression of these novel genes, we further conducted a double ISH experiment using NvNcol-3 as a cnidocyte marker. An overlapping expression was observed, demonstrating that these novel genes, indeed, exhibit cnidocyte-specific expression profiles (Additional file 7: Figure S6).

### Annotation of cnidocyte-associated biochemical pathways

Functional annotation of differentially expressed genes revealed that a large number of upregulated genes in cnidocytes (46% and 36% in positive and super-positive samples, respectively) are taxonomically restricted. Further, gene enrichment analyses enabled the identification of functional categories that are significantly enriched or depleted in positive and super-positive cell populations, providing novel insights into the biochemical pathways of these enigmatic cells (Fig. 3e; Additional file 8: Figure S7; Additional file 9: Figure S8). The enrichment of the terms related to the extracellular matrix (Fig. 3e; Additional file 8: Figure S7; Additional file 9: Figure S8) supports a strong evolutionary link between the structural components of the cnidocyst and the constituents of the extracellular matrix [24]. Surprisingly, despite cnidocytes being specialized neural cells known for expressing several ion channel subtypes [25–27], a depletion of genes associated with ion channels, such as “cation transport,” “sodium ion channels,” and “potassium ion channels” was found (Fig. 3e; Additional file 8: Figure S7; Additional file 9: Figure S8).

### Recruitment of a stress-response complex into nematogenesis

**Fig. 6:**
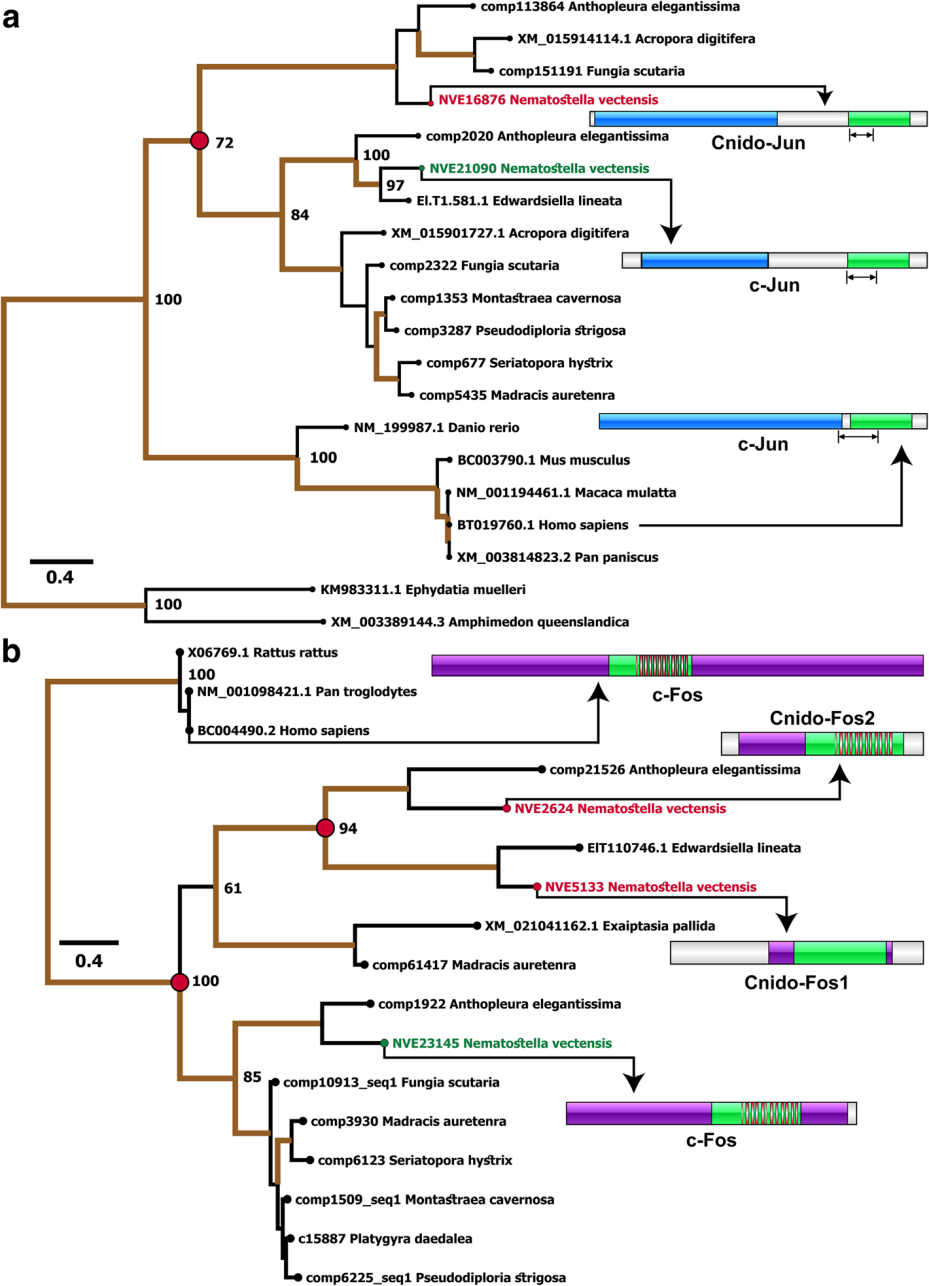
The deep origin of novel cnidocyte-specific transcription factors in Cnidaria. This figure depicts the phylogenetic histories of (**a**) the c-Jun and **b** c-Fos family of proteins in Cnidaria. Duplication events leading to the origin of Cnido-Jun and Cnido-Fos1—the two cnidocyte-specific transcription factors identified in this study (labeled in red font)—and c-Jun and c-Fos (indicated in green font) in Hexacorallia are shown in red circles. Branches with node support ≥ 70 are shown in thick brown lines, and the bootstrap values for the major nodes are indicated. Domain organization of these proteins are also depicted, where the Jun-like domain, the bZIP domains, and the c-Fos-related domains are indicated in blue, green, and purple, respectively. The DNA binding domain is marked by black arrows, while the dimerization domains are depicted in red bars

By retrieving homologs from the genomes and transcriptomes of a diversity of cnidarian species, we reconstructed the phylogenetic histories of two transcription factors that were found to be specifically upregulated in cnidocytes (*NVE16876* and *NVE5133*; Fig. 6). We named these transcription factors Cnido-Jun (4 and 1.5 log_2_ fold upregulation in positive and super-positive cells, respectively) and Cnido-Fos1 (5 and 4.3 log_2_ fold upregulation in positive and super-positive cells, respectively) to distinguish them from their paralogs encoding proto-oncogenic transcription factors c-Jun and c-Fos. These transcription factors are known to dimerize into an activation protein-1 (AP-1) complex, which is involved in various stress responses in Bilateria and Cnidaria [28–30]. We show that Cnido-Jun and Cnido-Fos1 protein-coding genes originated via gene duplication in the common ancestor of Hexacorallia—sea anemones and stony corals (Fig. 6a, b). However, only in sea anemones, the c-Fos gene underwent an additional round of duplication and led to the origination of Cnido-Fos2 (*NVE23145*) but was not identified as differentially expressed in a significant manner. Domain scanning of these transcription factors revealed cnidarian-specific insertions between the Jun and bZIP domains of the c-Jun proteins—the latter is required for DNA binding (Fig. 6). In a complete contrast, we found insertions in bilaterian c-Fos proteins that were missing in their cnidarian counterparts. Interestingly, Cnido-Fos1 was the only protein that had different residues than those implicated in the heterodimer formation (Fig. 6; Additional file 10: Figure S9), which may suggest that this derived protein binds to novel partner proteins.

**Fig. 7:**
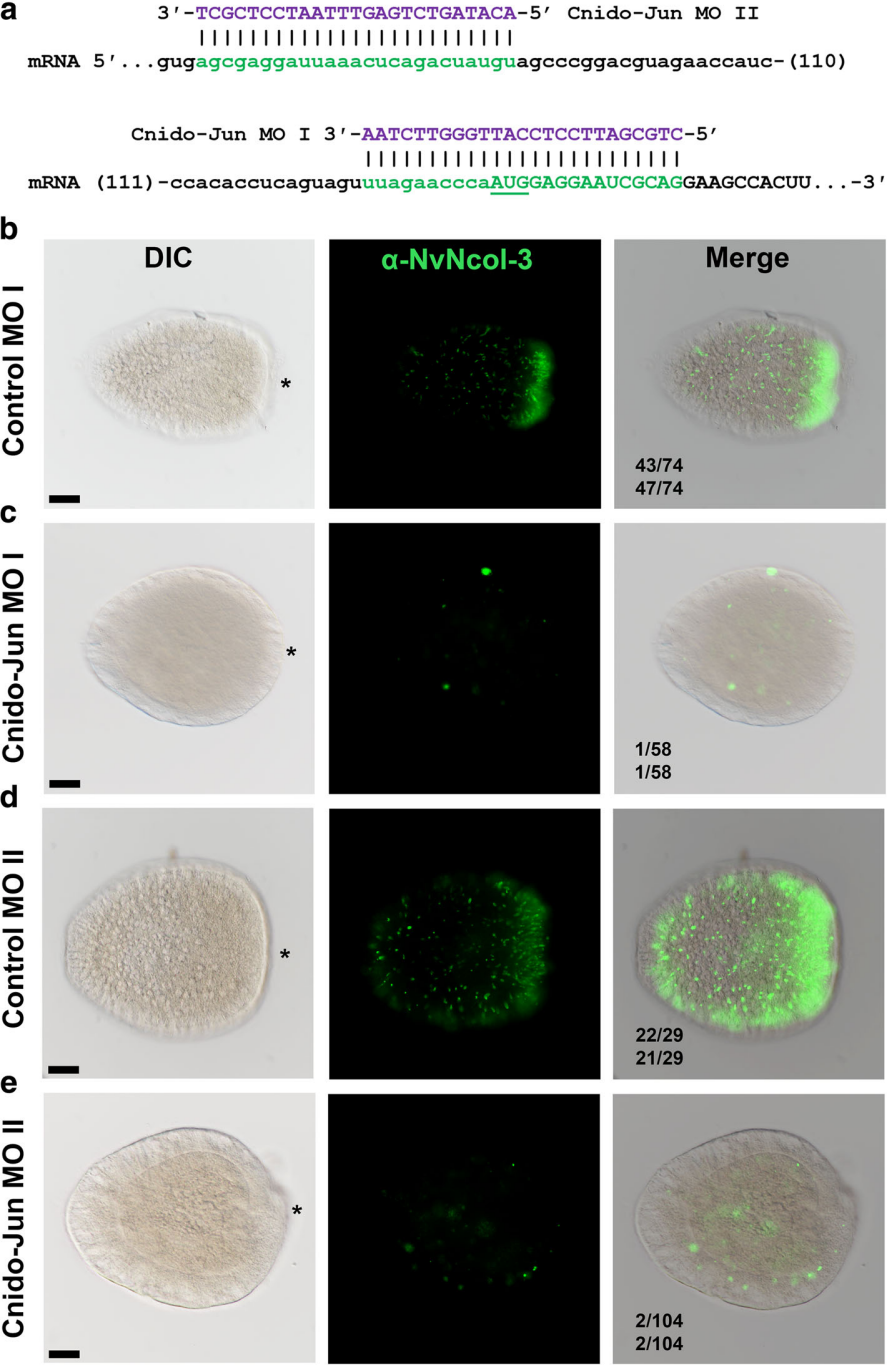
Knockdown of Cnido-Jun results in decreased NvNcol-3 expression. (**a**) Morpholino antisense oligo design. The MO bases appear in purple, and the sequence of its binding site in the mRNA appears in green. The AUG translation start site is underlined. **b** Planulae that were injected as zygotes with control MO exhibit normal expression of NvNcol-3. **c** In contrast, planulae injected as zygotes with Cnido-Jun MO exhibit abnormal expression of NvNcol-3, missing the dense expression domain in the aboral pole and most positive cells lack the typical elongated morphology of NvNcol-3-expressing cells. Panels (**d**) and (**e**) present the effects of a second non-overlapping MO against Cnido-Jun and the control MO that was injected in parallel. Oral pole in panels (**b**–**e**) are indicated by asterisk, and scale bars are 50 μm. The numbers of embryos exhibiting a normal NvNcol-3 phenotype out of each injected group based on the two indexes described in the text appear below each merged picture

To understand whether Cnido-Jun, which exhibits cnidocyte-specific expression, plays a significant role in nematogenesis, we manipulated its expression in *Nematostella* embryos. Using injection of morpholino antisense oligo (MO), a well-established method in *Nematostella* [31–33], we knocked down the expression of Cnido-Jun and assayed cnidocyte development by immunostaining with α–NvNcol-3 at the planula stage (Fig. 7). Strikingly, animals injected with the Cnido-Jun MO had far lower expression of NvNcol-3, and the cells positive for α–NvNcol-3 did not exhibit the typical elongated shape observed in the control MO animals (Fig. 7). Moreover, the very dense population of α–NvNcol-3-positive cells typically observed in the oral pole of the planulae was strikingly missing from the animals injected with Cnido-Jun MO (Fig. 7). To verify the specificity of the effect, we repeated these experiments with a second non-overlapping MO against Cnido-Jun. We counted two different criteria that represent normal cnidogenesis in *Nematostella*: (i) presence of high NvNCol-3 expression in a ring-shaped domain around the oral pole of the planula. In this count only one of 58 animals in the Cnido-Jun MO-injected group exhibited expression in this domain, whereas 43 of 74 animals exhibited this expression domain in the control MO-injected group, and (ii) presence of elongated NvNCol-3-positive cells that make at least 20% of all cells positive for this marker in the planula. In this count, only one of 58 animals in the Cnido-Jun MO-injected group exhibited such proportion, whereas 47 of 74 animals exhibited this proportion in the control MO-injected group. For the second non-overlapping MO, the observed morphology and percentage of affected animals counted by the same indices were highly similar, providing strong support for the specificity of the assay (Fig. 7).

## Discussion

Among the large number of upregulated genes detected within the positive cell population (Fig. 3c, d; Additional file 3: Figure S3a), many were known cnidocyte marker genes, including toxin (*NEP-3*, *NEP-3-like*, *NEP-4*, and *NEP-5*) and structural protein-coding genes (*NvNcol-1* and *NvNcol-4*). At the same time, there was downregulation of certain neuronal marker genes (*FMRFamide* and *ELAV*) that are known to lack expression in cnidocytes (Additional file 4: Figure S4; Additional file 6: Table S1). Though there was consistency with regard to the downregulation of *FMRFamide* and *ELAV*, we did not identify the upregulation of the aforementioned cnidocyte marker genes in the super-positive cell population (Additional file 5: Figure S5), which was enriched with mature cnidocytes. This can be explained by the fact that in mature cnidocytes, the capsule, which is a very tight polymer of various peptides and proteoglycans, is already formed and the secretion of structural proteins is no longer required and is most probably wasteful. As a result, the expression of such genes diminishes with the maturation of cnidocytes. This is clearly evident when examining the expression of memOrange2 across various cell types. In our reporter line, memOrange2 was integrated into the genomic locus of *NvNcol-3*—the gene coding a vital structural protein of the capsule (Fig. 1a). The expression of memOrange2 in the positive cell population was relatively higher than that in the negative cell population, but it dropped significantly in the super-positive population, where it was identified as a highly downregulated gene (− 1.67 log_2_ fold change; *p* value 0.0005) (Additional file 4: Figure S4; Additional file 5: Figure S5). This clearly demonstrates that the expression of structural proteins drops significantly with the maturation of capsules in the fully developed cnidocytes. Since the development of the mature capsule impedes the migration of secreted toxins, a similar pattern of expression can be expected for toxin-coding genes at this stage of development. As explained in the following section, these results can also be attributed to the inherent dynamics of gene expression across developing cnidocytes.

It should be noted that because the secreted memOrange2 protein requires 4.5 h at 37 °C for t_1/2_ maturation [34], a smaller fraction of cnidocytes in the very earlier stages of development may contain an immature non-glowing memOrange2 and, therefore, would get sorted into the negative population. As a result, despite the increased expression in positive cells in comparison to the negative cells, memOrange2 was not identified as a differentially expressed transcript. As explained in the “Results” section, the temporal variation in transcription and translation of memOrange2 could also contribute to this effect (Fig. 4i). However, the robustness of our experimental approach in isolating cnidocytes for generating cell type-specific transcriptomic profiles is strongly supported by multiple lines of evidence: (i) the significant upregulation of cnidocyte-specific markers (ranging between 1.8 and 4.6 log_2_ fold change difference) and the downregulation of neuronal markers in positive cells (Additional file 4: Figure S4), (ii) microscopic examinations of isolated cells (Fig. 2b, d; Additional file 1: Figure S1), (iii) in situ hybridization experiments for the ten highly upregulated genes in cnidocytes (Fig. 5), and (iv) the functional annotation of upregulated transcripts (Additional file 8: Figure S7; Additional file 9: Figure S8). Moreover, the microscopic examination of the positive cell populations revealed many cnidocytes in a very early stage of maturation (Additional file 1: Figure S1). These cells can clearly be seen to contain undeveloped capsules and multiple secretory vesicles carrying premature cnidocyst components, which indicate early stages of nematogenesis when the capsule starts to form by massive Golgi secretions [1]. Many were even completely missing capsules but contained only memOrange2-filled vesicles (Additional file 1: Figure S1), suggesting that our approach can capture cnidocytes in early stages of maturation. However, the transgenic approach might have some limitations in detecting genes expressed very early in cnidocyte development since the reporter fluorescence is by nature temporally lagging behind the expression of these genes (i.e., the time required for *memOrange2* translation and maturation). We speculate that this is the reason why some genes previously reported to be involved in early nematogenesis such as PaxA and Mef2 were not recovered in our analysis [23, 35].

The dramatic difference we discovered in the expression profiles of certain genes in the positive and super-positive cell populations can be further attributed to the inherent dynamics of transcription and translation across various stages of cnidocyte maturation. This was revealed by a combination of ISH and immunostaining experiments, where we co-localized the transcripts and proteins encoded by *memOrange2* or *NvNcol-3* genes and is in agreement with previous results [23]. With this approach, we detected three different populations of cnidocytes: (i) cnidocytes in the very early stages of maturation that only contained abundant transcripts but no proteins, suggesting that they were yet to develop a capsule; (ii) cnidocytes with high levels of both transcripts and proteins, suggesting that these were developing cells but contained an immature capsule; and (iii) mature cnidocytes with fully developed capsules that only contained proteins and completely lacked transcripts for *memOrange2* and *NvNcol-3* genes (Fig. 4). This supports our hypothesis that transcription of toxin and structural protein-coding genes drastically falls in mature cnidocytes with the complete formation of the tightly polymerized capsule. Thus, we reveal spatiotemporal dynamics of transcription and translation of certain genes within cnidocytes (Fig. 4).

Our experimental and bioinformatic approaches facilitated the identification of a large number of cnidocyte-specific genes, many of which were taxonomically restricted (46% and 36% in positive and super-positive samples, respectively; Additional file 6: Table S1), highlighting a paucity of knowledge regarding the biology of cnidocytes. We identified nine genes that were significantly upregulated in positive cells (log_2_ fold change in the range of 2.8 to 5) and were not known to be specifically expressed in cnidocytes. The localization of expression in ISH experiments strongly suggested that all ten genes exhibited cnidocyte-specific expression (Fig. 5), albeit with a spatiotemporal variation. For example, three of these genes encoded protein disulfide isomerases (*NVE15732*, *NVE15733*, and *NVE26200*), which probably mediate the folding of cysteine-rich toxins and structural proteins within cnidocytes that usually require the formation of multiple disulfide bridges for attaining the proper conformation and for carrying out their biological function [1, 24, 36]. Each of these enzymes exhibited distinct expression profiles (Fig. 5), which is suggestive of functional specialization of disulfide isomerases within the different types of cnidocytes. Similarly, the expression of the NOWA-like gene was limited to a smaller population of cnidocytes in the oral region of the planula and the tentacles of the primary polyp. In *Hydra*, NOWA has been implicated in the formation of nematocyst [37]. If this protein serves a similar structural function in the cnidocytes of *Nematostella*, then the documented differences in expression profiles are suggestive of distinct structural makeup of different cnidocyte populations.

Interestingly, the expression of a calmodulin (*NVE22513*), a highly conserved protein known to bind Ca^2+^ ions [38], was only restricted to certain regions of the tentacles in the primary polyp (Fig. 5). Fluids of the mature cnidocytes are known to contain high concentrations (ca. 500–600 mmol kg^−1^ wet weight) of Ca^2+^ ions that are bound by certain proteins in the undischarged state [39], and the dissociation of Ca^2+^ ions from these partner proteins has been implicated in the rapid discharge of cnidocysts. Thus, the restricted expression profile of calmodulin points to the functional specialization of Ca^2+^ ion-binding proteins in different types of cnidocytes. Thus, the identification of numerous novel cnidocyte-specific genes has advanced our knowledge regarding the biology and development of cnidocytes.

Our analyses enabled the discovery of Cnido-Jun and Cnido-Fos1, two novel cnidocyte-specific transcription factors that share very high sequence similarity with the c-Jun and c-Fos family of proteins. c-Jun and c-Fos are known to constitute the AP-1 early-response transcription factors that are associated with stress, infection, cytokines, and other stimuli [29]. In *Nematostella*, a single *c-Jun* (*NVE21090*) and a single *c-Fos* (*NVE23145*) have been identified as players in stress response [28, 40], whereas expression levels of *Cnido-Jun* and *Cnido-Fos* (referred to as *Jun2* and *Fos2*, respectively, in one of the previous studies) were found to be insensitive to induced stress conditions. Results of our phylogenetic analyses revealed that *Cnido-Jun* and *Cnido-Fos1* genes originated via duplication events in the common ancestor of hexacorallians (sea anemones and corals), approximately 500 mya [41] (Fig. 6). These two genes were found to be significantly upregulated within cnidocytes, ranging between 4 and 5 log_2_ fold upregulation, when compared to the negative cells. However, unlike Cnido-Jun and Cnido-Fos1, the expression of the c-Jun and c-Fos stress response transcription factors was not upregulated in cnidocytes when compared to other cells. Therefore, it is very likely that the cnidocyte-specific Cnido-Jun and Cnido-Fos1 proteins, which might dimerize to form a “Cnido-Ap1” complex within cnidocytes, have been recruited into a function other than stress response, particularly a function related to nematogenesis.

To understand the role of these unique transcription factors within cnidocytes, we knocked down the expression of Cnido-Jun in *Nematostella* embryos. As this manipulation resulted in strong decrease in NvNcol-3 expression and defective morphology of the stained cells (Fig. 7), the results strongly suggest that *NvNcol-3*, a gene encoding one of the better characterized nematocyst capsule components, is either a direct or indirect target of Cnido-Jun and its partner protein(s).

The functional annotation of upregulated genes in positive and super-positive cell populations supported the earlier findings in *Hydra* showing that a significantly large proportion of cnidocyte-specific genes in Cnidaria are taxonomically restricted [10]. Gene enrichment analyses facilitated the discovery of significantly enriched or depleted annotation categories in the positive and super-positive cell populations, providing the first insights into the biochemical pathways of these peculiar cell types (Fig. 3e; Additional file 8: Figure S7; Additional file 9: Figure S8). A strong enrichment of annotations related to the hydrolytic activity was noted in the positive and super-positive cells, which can be explained by the abundant presence of proteolytic and hydrolytic enzymes in cnidocytes that are responsible for processing cnidocyst structural components, as well as for exerting toxicity in prey or predatory animals [8, 19, 42]. Annotation terms related to the extracellular matrix, such as the “basal lamina” (Fig. 3; Additional file 8e: Figure S7; Additional file 9: Figure S8), were found to be enriched in positive and super-positive cells. This supports the proposed evolutionary link between cnidocyst structural components and the extracellular matrix constituents [24]. Interestingly, even though cnidocytes are specialized neural cells that are known to express several subtypes of ion channels [25–27], a depletion of annotation terms related to ion channels was identified (Fig. 3e; Additional file 8: Figure S7; Additional file 9: Figure S8). This can be explained by the fact that many subtypes of ion channels are known to be specifically expressed in certain other cell types, such as neurons and muscles, but not in cnidocytes [27, 43–45]. The surprising enrichment of annotation terms like “cytokine-mediated signaling pathway,” “cellular response to insulin stimulus,” and “regulation of cell motility” in cnidocytes further highlights the presence of several uncharacterized biochemical pathways that might be relevant to cnidocytes. Thus, several specialized ion channels and biochemical pathways appear to be unique to cnidocytes.

## Conclusions

We conclude that nematogenesis or the differentiation of neuronal precursor cells into cnidocytes is a highly dynamic and multistep process that is accompanied by complex shifts in the transcriptional and translational profiles of differentiating cnidocytes. We highlight a drastic reduction in the transcriptional profiles of toxins and structural proteins within the mature cnidocytes that have a fully developed capsule. We show that a significantly large number of upregulated genes and biochemical pathways within cnidocytes are yet to be characterized, and thus, there is a remarkable paucity of knowledge regarding the biology of cnidocytes. This study led to the identification of numerous novel protein-coding genes that show cnidocyte-specific expression. Most of them surprisingly exhibited spatiotemporal variation in expression and indicated the presence of a large population of uncharacterized cnidocytes. We also identified multiple duplication events that led to the recruitment of a bilaterian stress response complex into cnidocytes and how it is significant for nematogenesis. Thus, we provide several fascinating insights into the biology, development, and the evolution of cnidocytes, which are also the first venom-injecting cells to originate within animals, nearly 600 mya.

## Methods

### Sea anemone culture

*N. vectensis* polyps were grown in 16‰ sea salt water at 17 °C. Polyps were fed with *Artemia salina* nauplii three times a week. Spawning of gametes and fertilization were performed according to a published protocol [46]. In brief, temperature was raised to 25 °C for 9 h and the animals were exposed to strong white light. Three hours after the induction, oocytes were mixed with sperm to allow fertilization.

### Transgenesis

For generating a transgenic line expressing memOrange2 under the native regulatory region of *NvNcol-3*, we microinjected a *Nematostella* zygote with a mixture that included guide RNA (250 ng/μl) of the sequence CAGUAGUUAGGGCAUCCCGG (as part of a transcript also carrying a tracrRNA), Cas9 recombinant protein with nuclear localization signal (500 ng/μl; PNA Bio, USA), and a donor plasmid that includes two homology arms (spanning positions 1,380,459-1,381,924 and 1,381,925-1,383,035 in scaffold 23 of the *N. vectensis* genome) spanning the *memOrange2* gene. This construct encodes a chimeric protein sequence that carries the first 32 amino acids of NvNcol-3, fused to the memOrange2 sequence, separated by a flexible linker sequence of 2 × (Gly-Gly-Ser) to allow proper folding of the fluorescent protein. The expression of the transgene in injected zygotes and embryos was monitored under a Nikon SMZ18 fluorescent stereomicroscope equipped with a Nikon Ds-Qi2 monochrome camera and Elements BR software (Nikon, Japan).

### Dissociation of tentacles and cell sorting

Tentacles of *Nematostella* polyps were dissociated using a combination of papain (2 mg/ml; Sigma-Aldrich: P4762), collagenase (2 mg/ml; Sigma-Aldrich: C9407), and pronase (4 mg/ml; Sigma-Aldrich: P5147) in DTT (1.3 M) and PBS (10 mM sodium phosphate, 175 mM NaCl, pH 7.4). The tentacles were incubated with the proteolytic cocktail at 22 °C overnight along with Hoechst stain (Sigma-Aldrich) to stain the nucleus. This was followed by the dissociation of tissues into single cells by flicking the tubes gently and centrifugation at 400×*g* for 15 min at 4 °C. The pellet was gently reconstituted in PBS. A small amount of the dissociated sample was subjected to microscopic examination to verify successful cell separation, while the remaining sample was used for fluorescence-activated cell sorting in a FACSAria III (BD Biosciences, USA) equipped with a 488-, 405- and 561-nm lasers and a 70-μm nozzle. Two distinct populations of cells were sorted and collected: (a) Hoechst-positive and memOrange2-negative cell populations and (b) Hoechst-positive and memOrange2-positive cell populations. The cells were directly collected into TRIzol® LS reagent (Thermo Fisher Scientific) in 3:1 reagent to sample ratio. The instrument was maintained at 4 °C throughout the procedure. Positive and negative cells were collected from the tentacles of three independent batches of animals that formed our biological replicates.

### RNA isolation and sequencing

Total RNA from the positive and negative populations was isolated using the TRIzol® LS reagent according to manufacturer’s protocol. Following isolation, the RNA samples were treated with Turbo DNase (Thermo Fisher Scientific), followed by re-extraction with the Tri-Reagent to remove DNase. RNA quality was assessed on a Bioanalyzer Nanochip (Agilent, USA), and only samples with RNA integrity number (RIN) ≥ 8.0 were considered for sequencing (all samples except POS7: 7.5 RIN). Sample library preparation for RNA sequencing was accomplished using the Illumina TruSeq RNA library protocol (mean insert size of 150 bp). The samples were sequenced on Illumina Nextseq 500 high output v2 platform (2 × 40 bp), which generated an average of 50 million paired-end reads per replicate. The Illumina BaseSpace pipeline was used for de-multiplexing and filtering high-quality sequencing reads. Additional quality filtering steps were performed using Trimmomatic v0.36 [47] to remove adapters, leading and trailing low-quality bases (below quality 3), very short reads (shorter than 20 bases), and reads with less than an average quality score of 20 using a sliding window of 4 bases. The quality of the preprocessed and the processed data was verified using FastQC [48].

### Differential gene expression and transcript annotation

Reads were aligned to the indexed genome of *Nematostella* [49] using STAR [50], followed by the quantification of gene expression with HTSeq-count v0.6 [51]. The *Nematostella* gene models used in all analyses were previously utilized in other studies [52, 53] and can be found at https://figshare.com/articles/Nematostella_vectensis_transcriptome_and_gene_models_v2_0/807696. Differential expression analyses were performed using two Bioconductor packages: DESeq v2.1 [54] and edgeR v3.16 [55]. Genes identified in concert by these two methods were considered as differentially expressed and were functionally annotated using Blast2go v4.1 [56] against Toxprot [57] and Swissprot (May 16, 2014) databases. A cutoff of 50 reads mapping to the negative sample, which filters out the lowly expressed genes, was employed to increase the stringency and accuracy of identifying differentially expressed genes.

### Whole mount in situ hybridization and immunostaining

Colorimetric ISH was performed following a published protocol [58]. Double ISH combining nitro-blue tetrazolium and 5-bromo-4-chloro-3′-indolyphosphate (NBT/BCIP) and FastRed (Roche, Germany) staining was also performed according to an established protocol [19]. Double fluorescent ISH (dFISH) was also performed according to published protocols [21, 22] with tyramide conjugated to Dylight 488 and Dylight 594 fluorescent dyes (Thermo Fisher Scientific, USA). In ISH and FISH, embryos older than 4 days were treated with 2u/μl T1 RNAse (Thermo Fisher Scientific) after probe washing, in order to reduce the background staining. Immunostaining was performed according to an established protocol [59], employing a commercially available rabbit polyclonal antibody against mCherry (Abcam, USA) that cross-reacts with memOrange2 and a custom-made guinea pig antibody against NvNcol-3 [14], generously provided by Suat Özbek (Heidelberg University). In experiments where ISH and immunostaining were combined, the ISH staining was performed with FastRed (Sigma-Aldrich). Stained embryos, larvae, and adult polyps were mounted in either Vectashield antifade medium (Vector Laboratories, USA) or 85% glycerol and visualized with an Eclipse Ni-U microscope equipped with a DS-Ri2 camera and an Elements BR software (Nikon, Japan) or with a Leica SP5 upright confocal microscope equipped with 405-, 488-, 561- and 594-nm lasers (Leica, Germany).

### Immunostaining of dissociated Nematostella cells

Immunostaining protocol for single cells was adapted from a published protocol for mammalian cell cultures [60]. PBS was added to the dissociated cells followed by centrifugation at 400×*g* for 3 min at 4 °C. The pellet was gently reconstituted in PBS and placed on a coverslip covered with 2 μl of Cell-Tak (Corning, USA) inside a 12-well plate at room temperature. After 4 h in which the cell suspension was left for attachment, it was fixed by 4% paraformaldehyde for 30 min at room temperature. The cell suspension was washed in PBS and blocked by 5% BSA in PBS solution for 20 min at room temperature. The primary antibody staining was employed for 2 h at room temperature by a rabbit polyclonal antibody against mCherry (Abcam) diluted 1:400. The primary antibody was then washed by PBS, and a custom-made guinea pig antibody against NvNcol-3 [14] diluted 1:400 and DAPI (Thermo Fisher Scientific) diluted 1:500 were added. The cell suspension was washed twice with PBS and mounted by adding 8 μl Vectashield antifade medium (Vector Laboratories) on positively charged slides. Details of all antibodies used in this work are available in Table 1.

**Table 1:** Antibodies used in this work

Name	Manufacturer	Catalog number	Lot number	RRID
Sheep anti-Digoxigenin-AP, Fab fragments	Roche/Sigma	11093274910	13680324	AB_514497
Sheep anti-Fluorescein-AP, Fab fragments	Roche/Sigma	11426338910	14973500	AB_514504
Sheep ant-Fluorescein-POD, Fab fragments	Roche/Sigma	11426346910	10715122	AB_840257
Sheep anti-Digoxigenin-POD, Fab fragments	Roche/Sigma	11207733910	11688900	AB_514500
Rabbit anti-mCherry antibody	Abcam	AB-ab167453	GR124924-35	AB_2571870
Guinea pig anti-NvNcol-3	Generously provided by Prof. Suat Özbek, Heidelberg University		Custom polyclonal	AB_2734725
Alexa Fluor 488 AffiniPure Donkey anti-Guinea Pig IgG (H+L)	Jackson ImmunoResearch	706-545-148	118980	AB_2340472
Alexa Fluor 594-AffiniPure Goat anti-Rabbit IgG (H+L)	Jackson ImmunoResearch	111-585-144	129771	AB_2307325

### Morpholino antisense oligo (MO) assays

Zygotes were microinjected with blocking-translation MOs of the sequences CTGCGATTCCTCCATTGGGTTCTAA (Cnido-Jun MO I) or ACATAGTCTGAGTTTAATCCTCGCT (Cnido-Jun MO II) matching the 5′ untranslated region and the beginning of the coding sequence of *Cnido-Jun* (Fig. 7a) and a control MO of the sequence CCTCTTACCTCAGTTACAATTTATA, which is predicted to not bind to any *Nematostella* transcript. MOs were designed and supplied by GeneTools (USA). The MOs were injected at 0.9 mM, and the injection mix contained 25 mM Dextran Alexa Fluor 488 (Thermo Fisher Scientific). The MO assay followed a published protocol [31]. The animals were fixed with ice-cold 4% paraformaldehyde for 45 min and incubated at 4 °C under gentle rotation, washed five times in PBS with 0.2% Triton X-100, and then stained with α–Ncol-3 following a published protocol [59].

### Phylogenetic analysis

Homologs of c-Jun and c-Fos transcription factors were retrieved using the tblastn search [61] against NCBI’s non-redundant nucleotide sequence database, *N. vectensis* genome [49], the EdwardsiellaBase [62], and various cnidarian transcriptomes [63]. The maximum likelihood analysis was utilized for the reconstruction of the molecular evolutionary histories of the c-Jun and c-Fos protein families. Trees were generated using PhyML 3.0 [64], and node support was evaluated with 1000 bootstrapping replicates.

## Additional files

Additional file 1: **Figure S1.** Maturation of cnidocytes. Images of the sorted positive and super-positive cells. The cells are observed in differential interference contrast (DIC) and mOrange filter under a fluorescent microscope. A merge of these two images is also provided. Scale bars are 10 μm. (TIF 6993 kb)

Additional file 2: **Figure S2.** Immunostaining of dissociated cells. Immunostaining with α-mCherry and α-NvNcol-3 of dissociated cells. Panels a, b, and c present single cells with vesicle-like structures positively stained for mCherry but not in DIC. d, A spirocyte positive for both antibodies. e, A nematocyst positive for both antibodies. Scale bars are 10 μm. (TIF 6584 kb)

Additional file 3: **Figure S3**. Heatmap of differentially expressed genes in positive and super-positive cells. Heatmaps of the top 250 upregulated and downregulated genes in positive (a) and super-positive (b) cnidocytes, relative to negative cells, across technical replicates. A color code for expression values (normalized log_2_ fold change rescaled between 2 and − 2), ranging from a gradient of maroon (downregulated) to blue (upregulated), is also provided. (TIF 530 kb)

Additional file 4: **Figure S4.** Cnidocyte-specific markers in positive cells. a Differential expression values of cnidocyte and neuronal markers for positive cells. b A heatmap of expression in the positive cell population, relative to negative cells, across technical replicates. A color code for expression values, ranging from a gradient of maroon (downregulated) to blue (upregulated), is also provided. (TIF 2050 kb)

Additional file 5: **Figure S5.** Cnidocyte-specific markers in super-positive cells. a Differential expression values of cnidocyte and neuronal markers for super-positive cells. b. A heatmap of expression in the super-positive cell population, relative to negative cells, across technical replicates. b A color code for expression values, ranging from a gradient of maroon (downregulated) to blue (upregulated), is also provided. (TIF 1903 kb)

Additional file 6: **Table S1.** Differentially expressed genes in positive and super-positive cell populations. (XLSX 759 kb)

Additional file 7: **Figure S6.** Double in situ hybridization of novel genes with *NvNcol-3*. Double in situ hybridization expression patterns of three novel cnidocyte-specific genes identified in this study are shown in late planulae. The novel genes were stained by NBT/BCIP (brownish-purple) while the *NvNcol-3* marker transcript was stained by FastRed (red). Examples for overlapping cells are indicated by purple arrow heads; cells which express the assayed gene but do not overlap with the cnidocyte marker are indicated by green arrow heads. Scale bar is 100 μm. (TIF 12135 kb)

Additional file 8: **Figure S7.** Biochemical pathways in positive cnidocytes. a Enrichment of GO terms in positive cnidocytes. b Upregulated GO terms for biological processes, cellular components, and molecular functions in the positive cell population. (TIF 813 kb)

Additional file 9: **Figure S8.** Biochemical pathways in super-positive cnidocytes. a Enrichment of GO terms in super-positive cnidocytes. b Upregulated GO terms for biological processes, cellular components, and molecular functions in the super-positive cell population. (TIF 973 kb)

Additional file 10: **Figure S9.** Sequence alignment of c-Fos protein family. Sequence identity is highlighted in shades of blue, while residues implicated in dimerization are marked by green arrowheads. Non-conserved dimerization residues in Cnido-Fos1 are shown in red. (TIF 951 kb)
