# Supplementary figures and images for: Cell type-specific expression profiling unravels the development and evolution of stinging cells in sea anemone
Source: BMC Biol. 2018 Sep 27;16:108. doi: 10.1186/s12915-018-0578-4 (PMC6161364; doi:10.1186/s12915-018-0578-4)

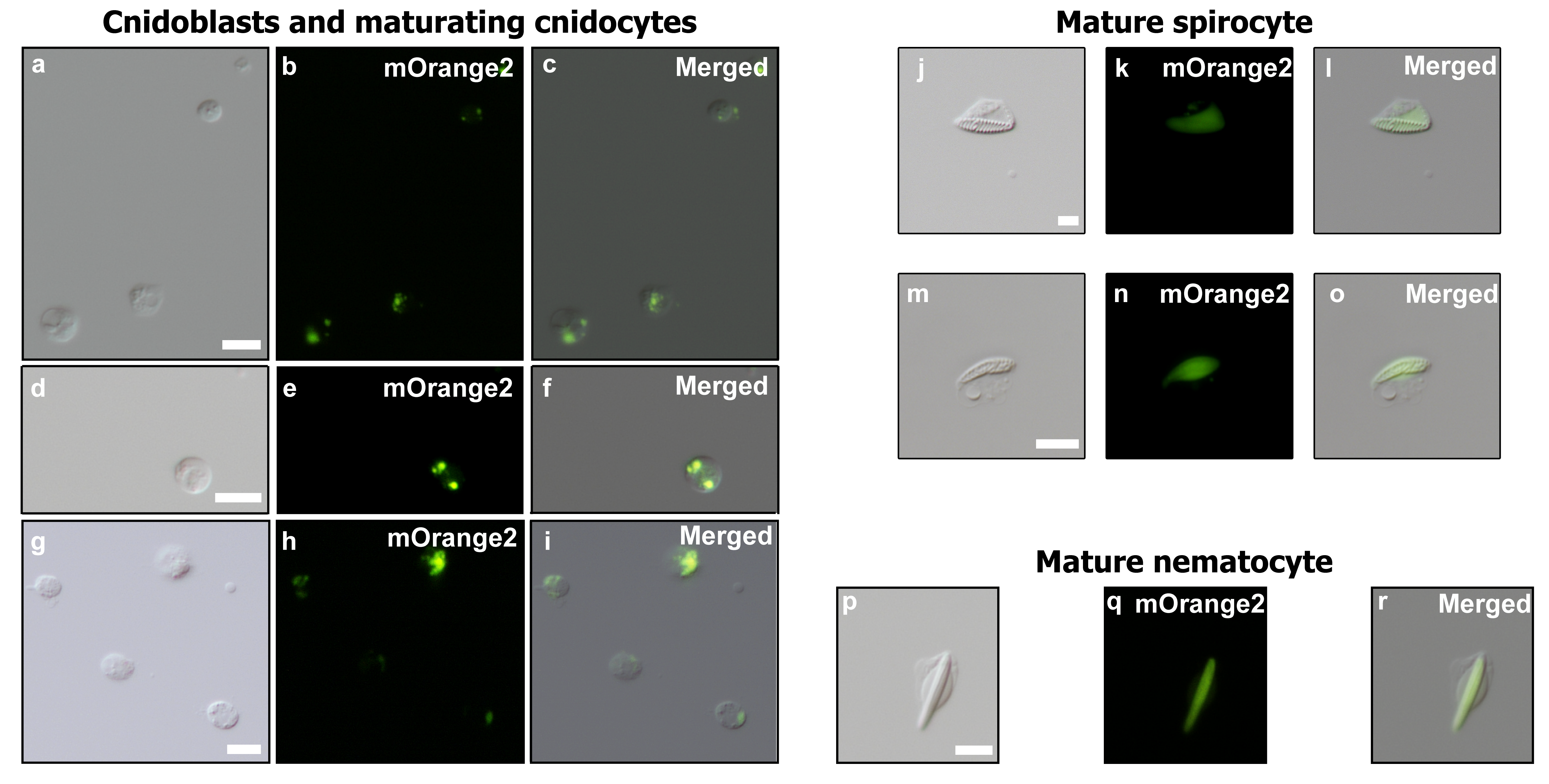

Supplement: Supplementary file 1 — Figure S1. Maturation of cnidocytes. Images of the sorted positive and super-positive cells. The cells are observed in differential interference contrast (DIC) and mOrange filter under a fluorescent microscope. A merge of these two images is also provided. Scale bars are 10 μm. (TIF 6993 kb) [file 12915_2018_578_MOESM1_ESM.tif]

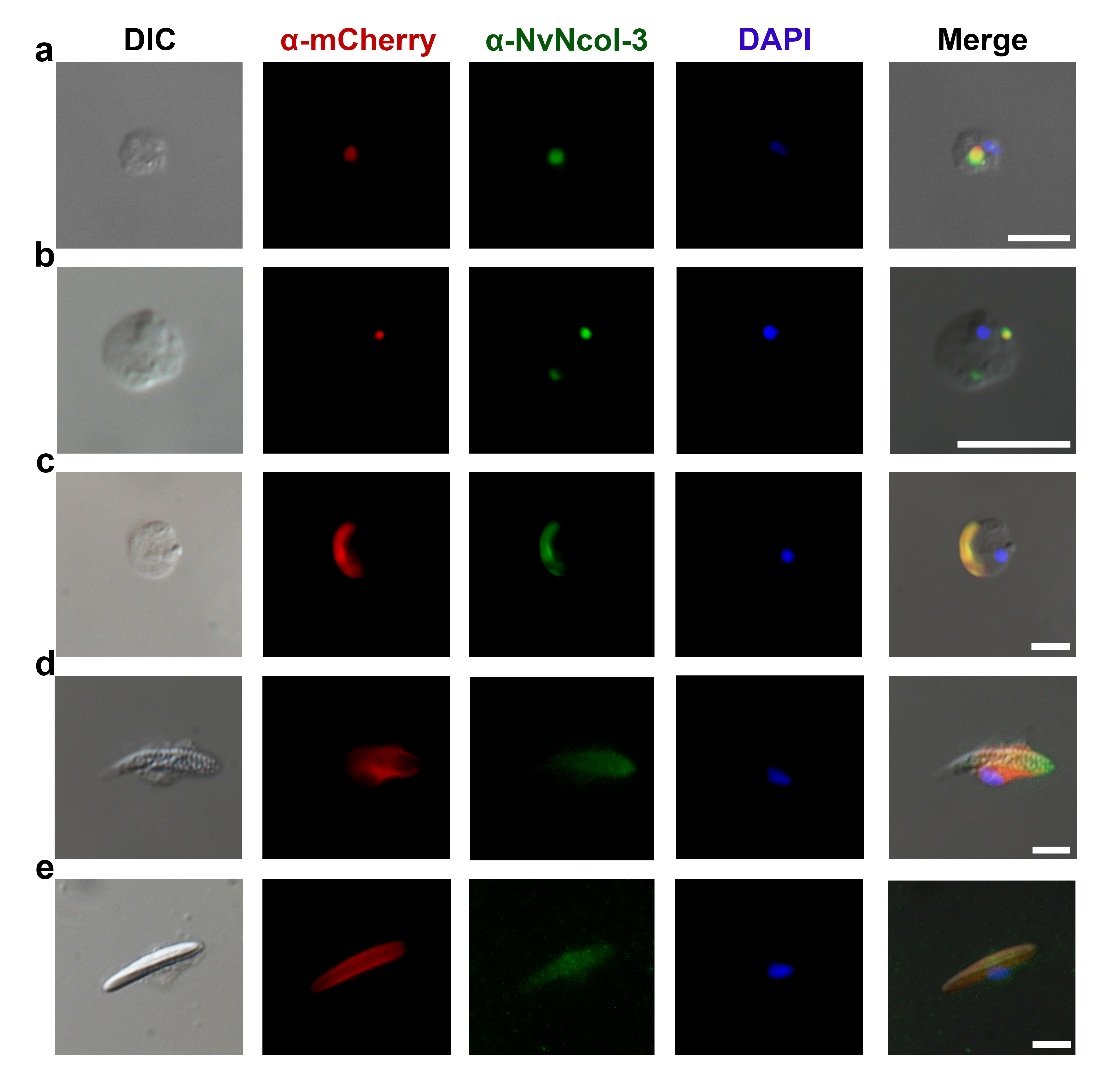

Supplement: Supplementary file 2 — Figure S2. Immunostaining of dissociated cells. Immunostaining with α-mCherry and α-NvNcol-3 of dissociated cells. Panels a, b, and c present single cells with vesicle-like structures positively stained for mCherry but not in DIC. d, A spirocyte positive for both antibodies. e, A nematocyst positive for both antibodies. Scale bars are 10 μm. (TIF 6584 kb) [file 12915_2018_578_MOESM2_ESM.tif]

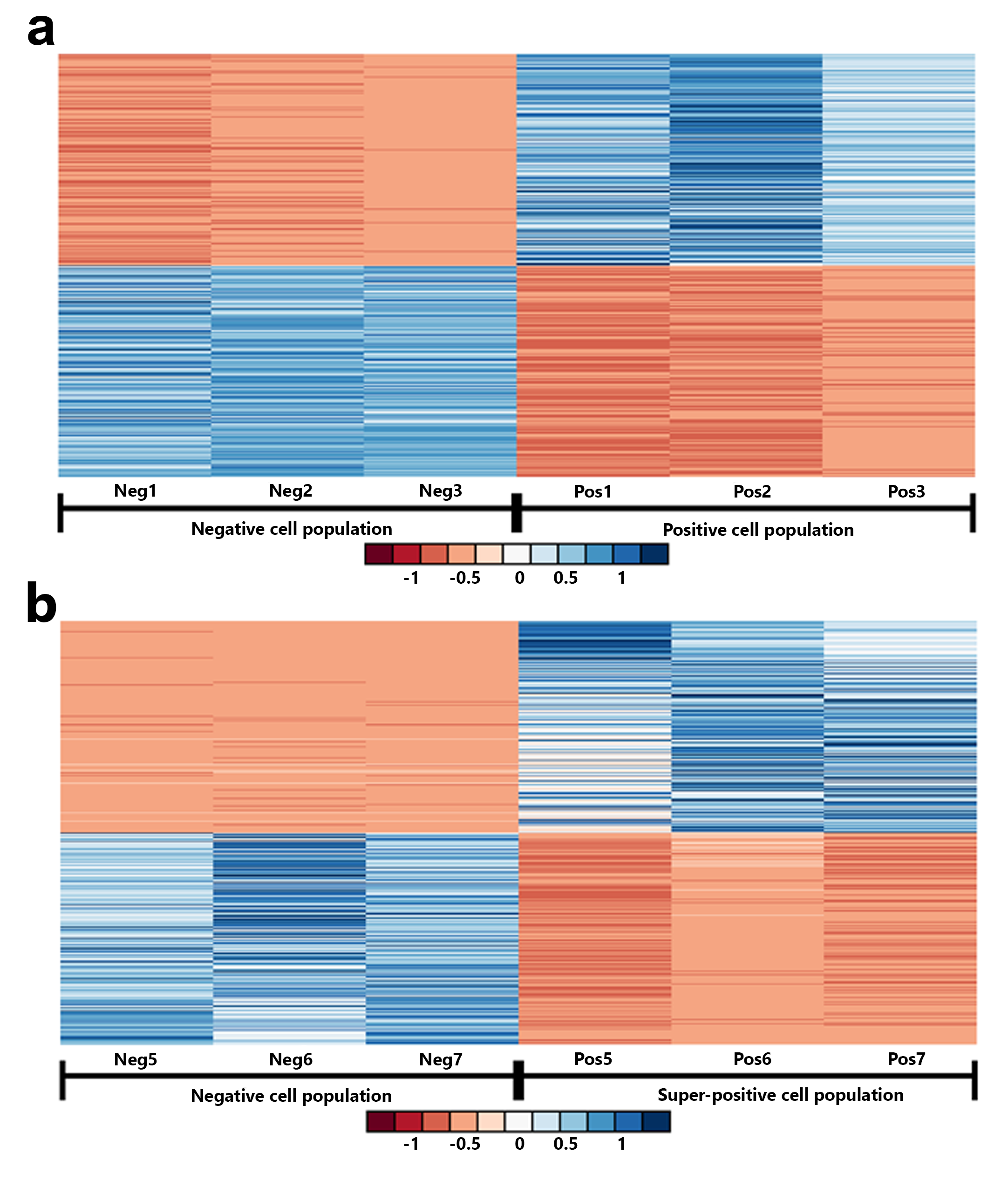

Supplement: Supplementary file 3 — Figure S3. Heatmap of differentially expressed genes in positive and super-positive cells. Heatmaps of the top 250 upregulated and downregulated genes in positive (a) and super-positive (b) cnidocytes, relative to negative cells, across technical replicates. A color code for expression values (normalized log2 fold change rescaled between 2 and − 2), ranging from a gradient of maroon (downregulated) to blue (upregulated), is also provided. (TIF 530 kb) [file 12915_2018_578_MOESM3_ESM.tif]

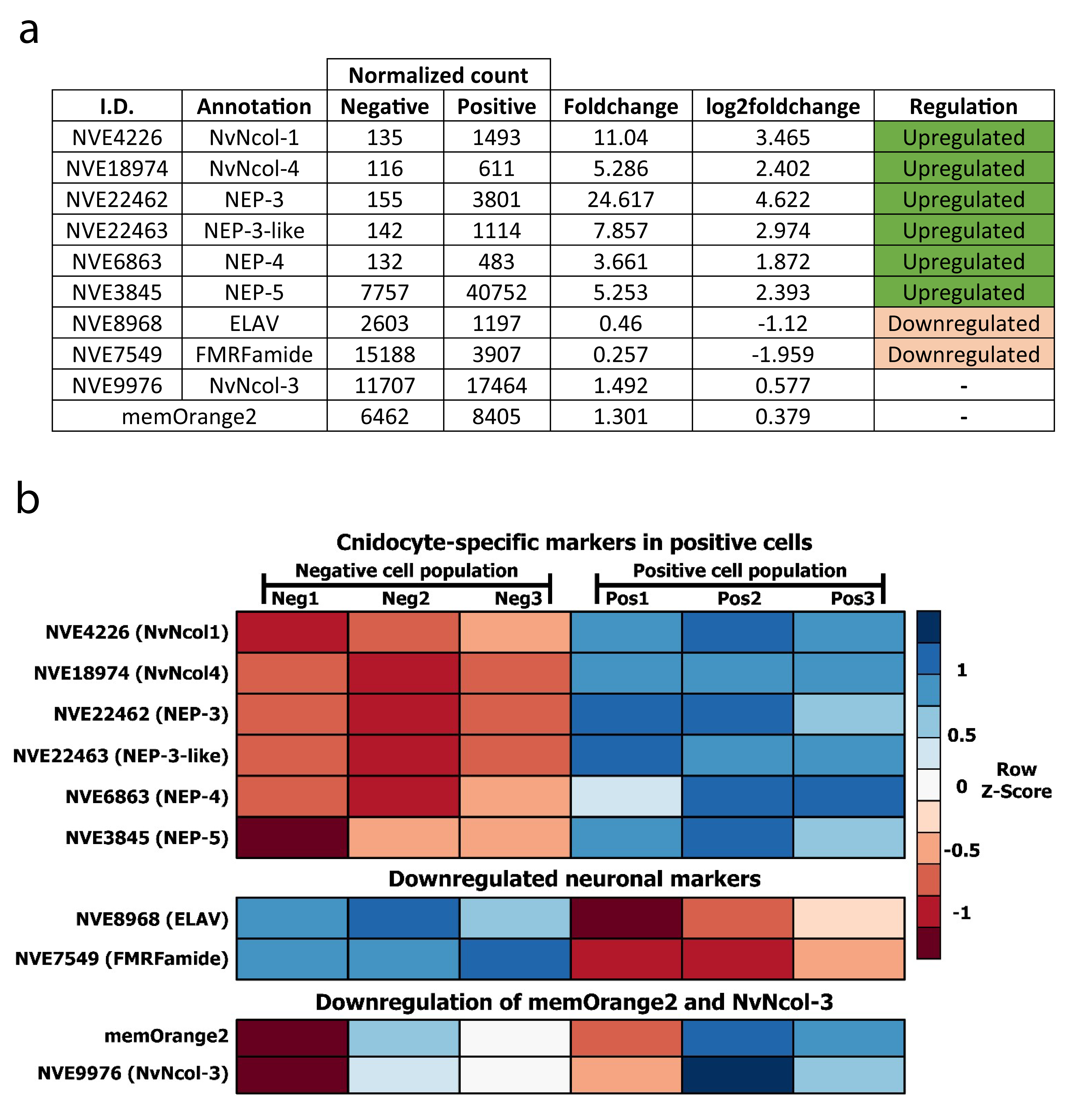

Supplement: Supplementary file 4 — Figure S4. Cnidocyte-specific markers in positive cells. a Differential expression values of cnidocyte and neuronal markers for positive cells. b A heatmap of expression in the positive cell population, relative to negative cells, across technical replicates. A color code for expression values, ranging from a gradient of maroon (downregulated) to blue (upregulated), is also provided. (TIF 2050 kb) [file 12915_2018_578_MOESM4_ESM.tif]

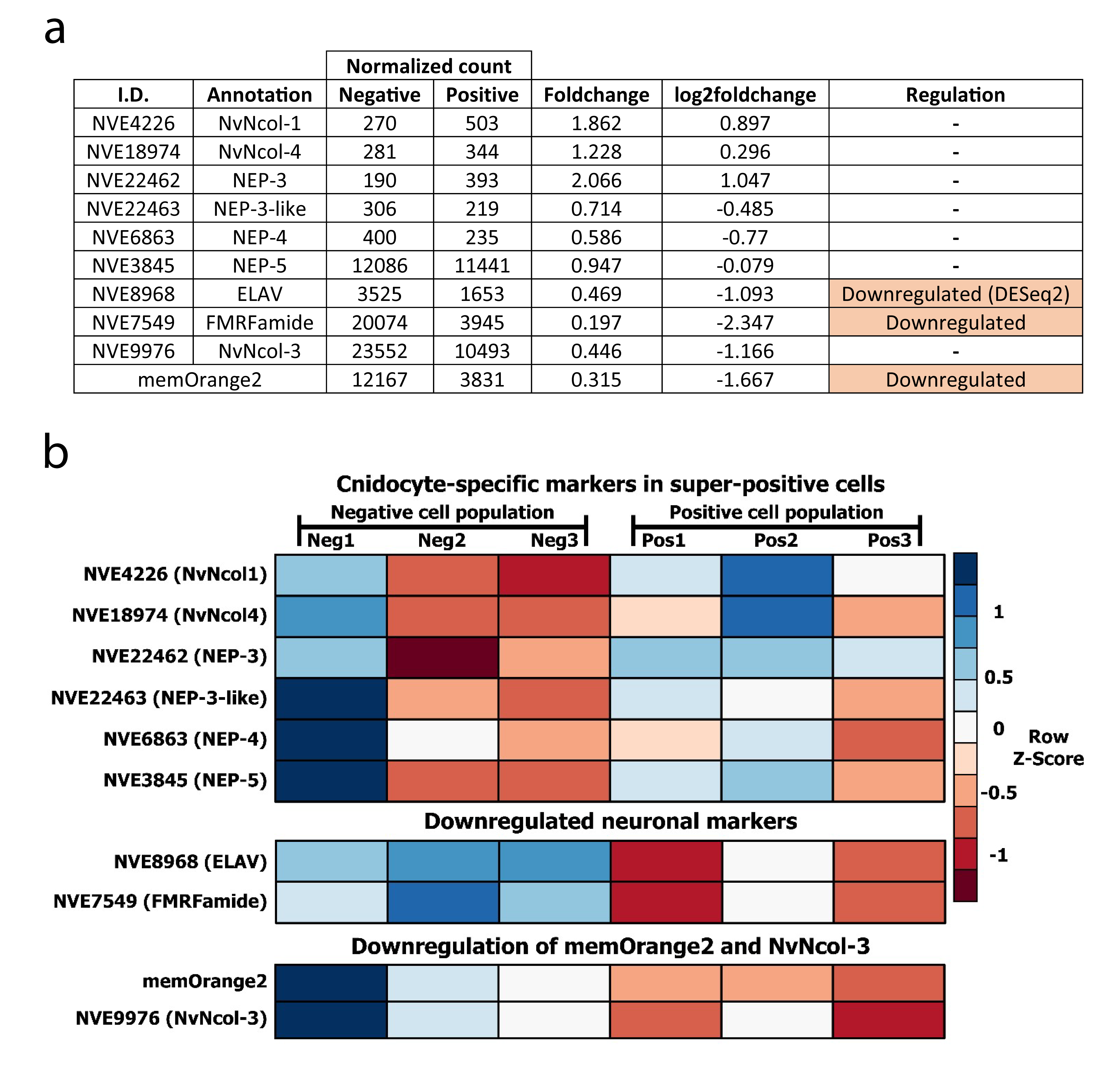

Supplement: Supplementary file 5 — Figure S5. Cnidocyte-specific markers in super-positive cells. a Differential expression values of cnidocyte and neuronal markers for super-positive cells. b. A heatmap of expression in the super-positive cell population, relative to negative cells, across technical replicates. b A color code for expression values, ranging from a gradient of maroon (downregulated) to blue (upregulated), is also provided. (TIF 1903 kb) [file 12915_2018_578_MOESM5_ESM.tif]

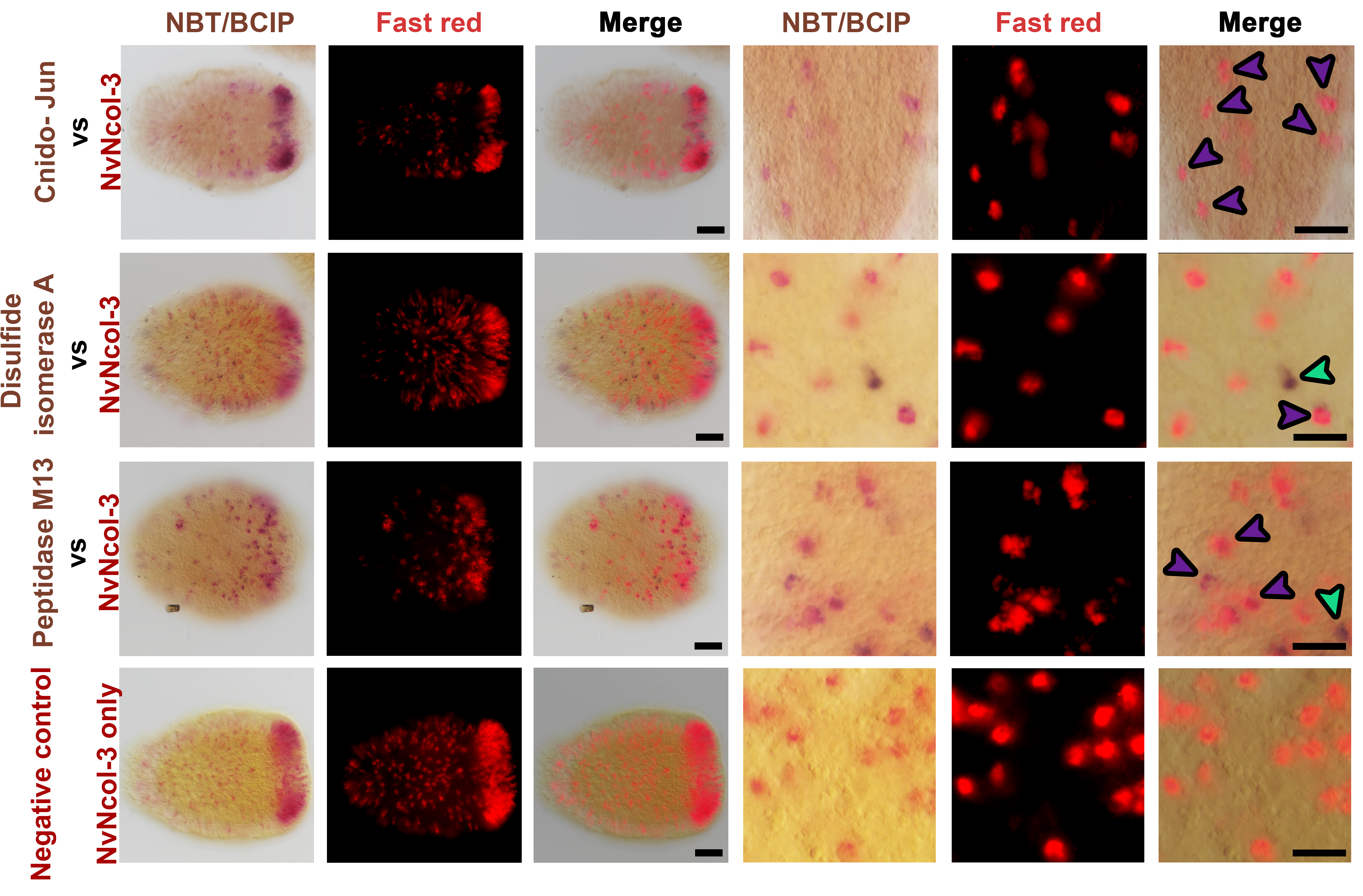

Supplement: Supplementary file 7 — Figure S6. Double in situ hybridization of novel genes with NvNcol-3. Double in situ hybridization expression patterns of three novel cnidocyte-specific genes identified in this study are shown in late planulae. The novel genes were stained by NBT/BCIP (brownish-purple) while the NvNcol-3 marker transcript was stained by FastRed (red). Examples for overlapping cells are indicated by purple arrow heads; cells which express the assayed gene but do not overlap with the cnidocyte marker are indicated by green arrow heads. Scale bar is 100 μm. (TIF 12135 kb) [file 12915_2018_578_MOESM7_ESM.tif]

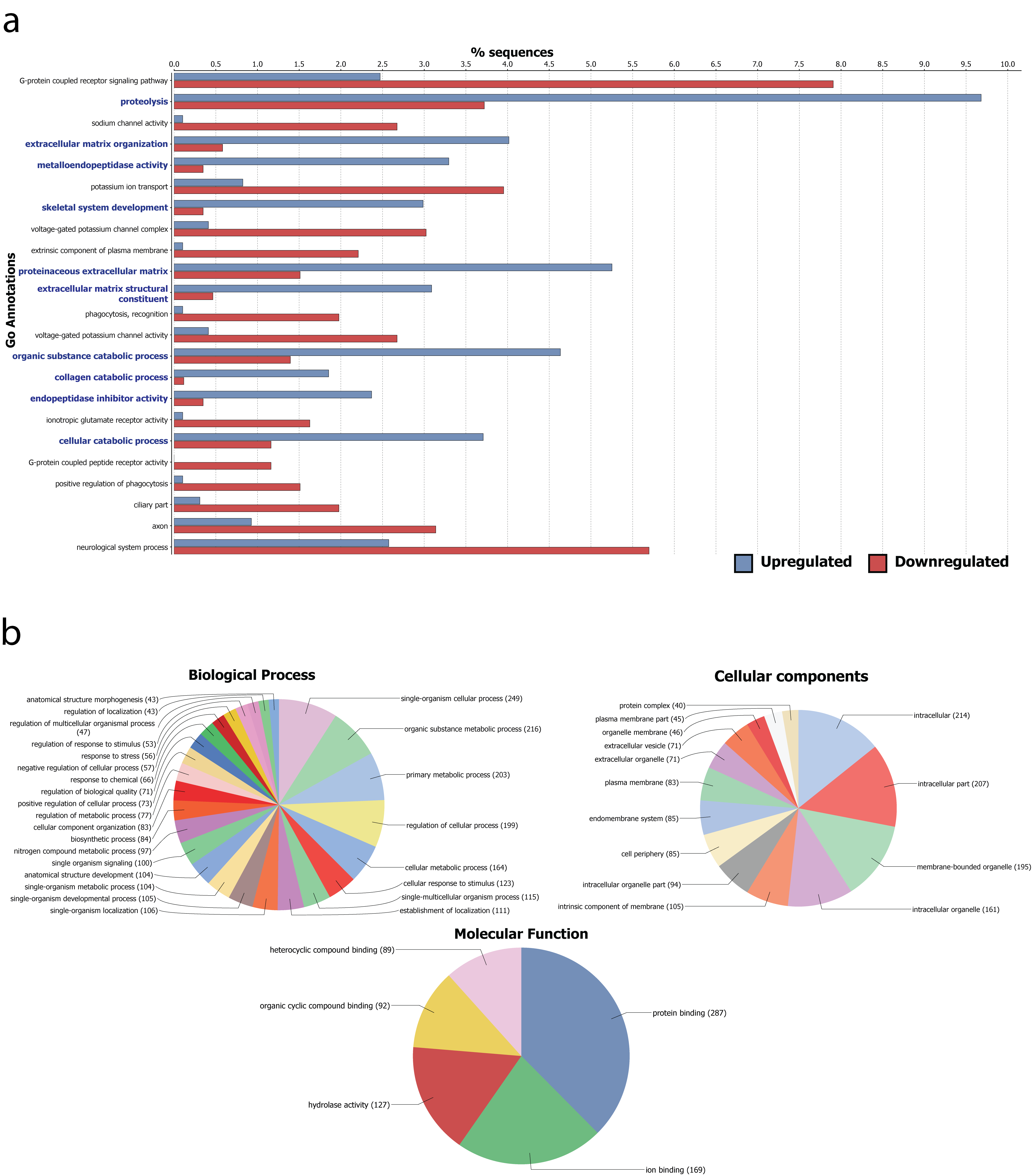

Supplement: Supplementary file 8 — Figure S7. Biochemical pathways in positive cnidocytes. a Enrichment of GO terms in positive cnidocytes. b Upregulated GO terms for biological processes, cellular components, and molecular functions in the positive cell population. (TIF 813 kb) [file 12915_2018_578_MOESM8_ESM.tif]

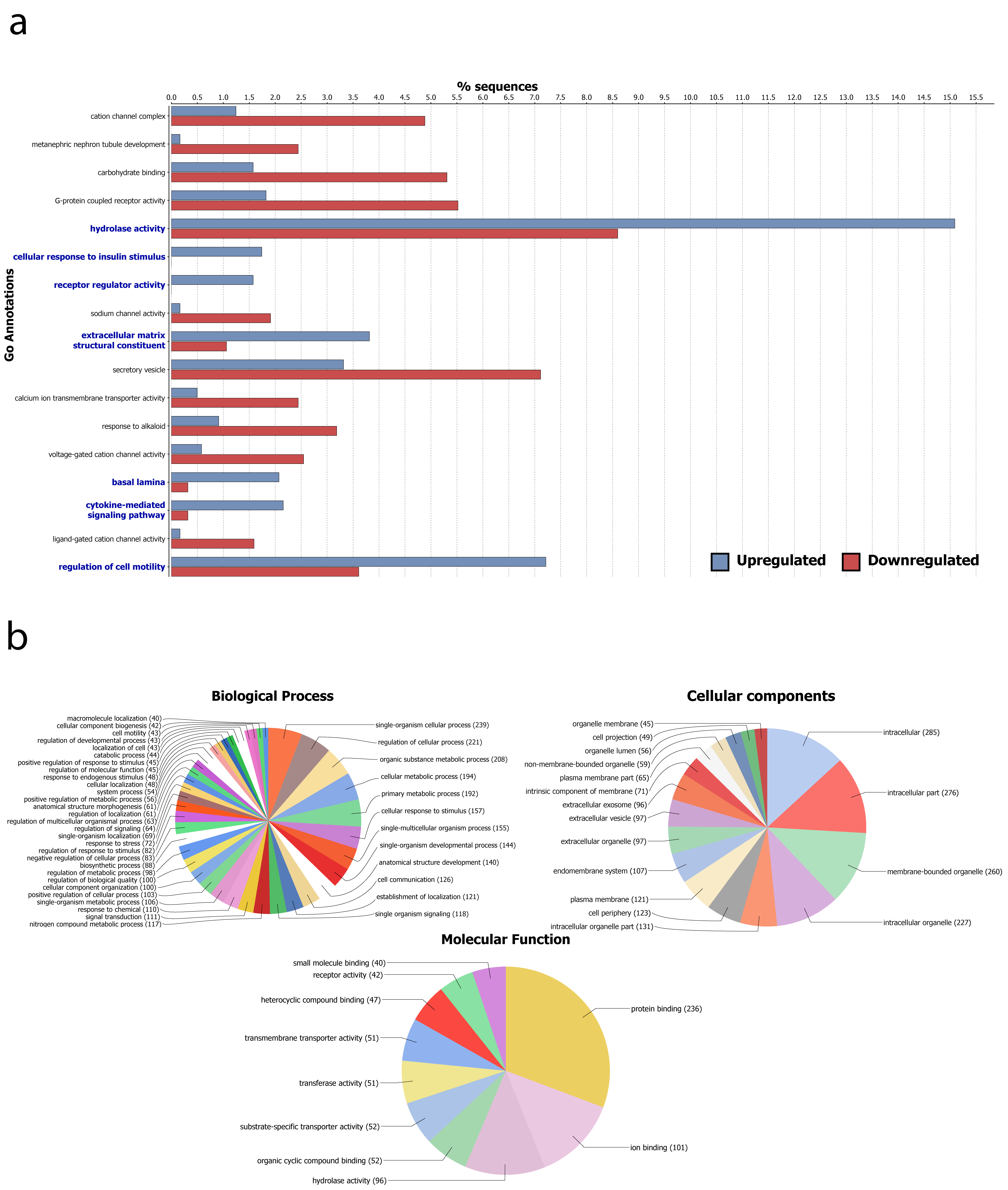

Supplement: Supplementary file 9 — Figure S8. Biochemical pathways in super-positive cnidocytes. a Enrichment of GO terms in super-positive cnidocytes. b Upregulated GO terms for biological processes, cellular components, and molecular functions in the super-positive cell population. (TIF 973 kb) [file 12915_2018_578_MOESM9_ESM.tif]

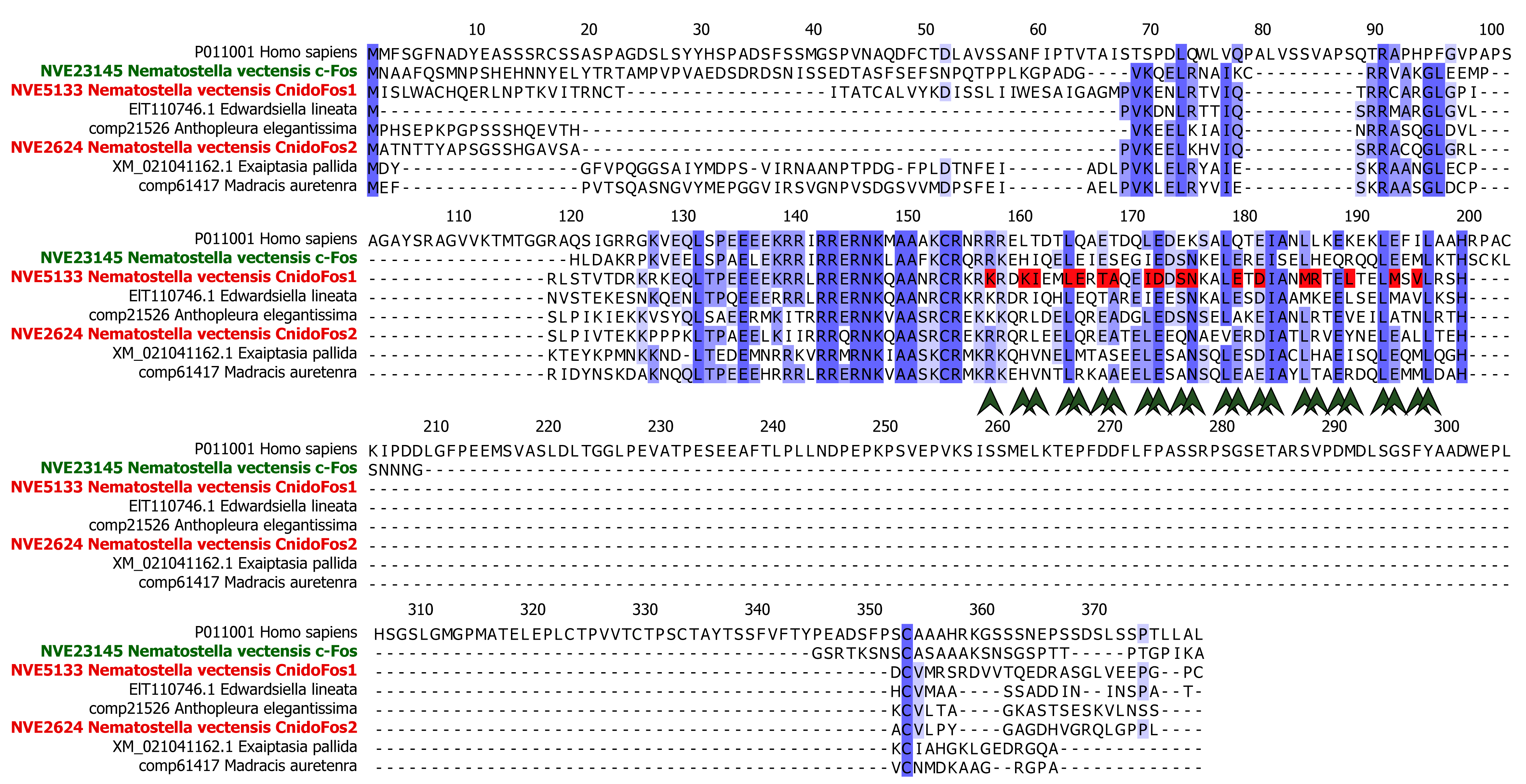

Supplement: Supplementary file 10 — Figure S9. Sequence alignment of c-Fos protein family. Sequence identity is highlighted in shades of blue, while residues implicated in dimerization are marked by green arrowheads. Non-conserved dimerization residues in Cnido-Fos1 are shown in red. (TIF 951 kb) [file 12915_2018_578_MOESM10_ESM.tif]
